# Supplementary material for: Linkage of voluntary medical male circumcision clients to adolescent sexual and reproductive health (ASRH) services through Smart-LyncAges project in Zimbabwe: a cohort study
Source: BMJ Open. 2020 May 4;10(5):e033035. doi: 10.1136/bmjopen-2019-033035 (PMC7223012; doi:10.1136/bmjopen-2019-033035)
Supplement: Author's manuscript [file bmjopen-2019-033035.draft_revisions.pdf]

BMJ Open is committed to open peer review. As part of this commitment we make the peer review history of every article we publish publicly available.

When an article is published we post the peer reviewers' comments and the authors' responses online. We also post the versions of the paper that were used during peer review. These are the versions that the peer review comments apply to.

The versions of the paper that follow are the versions that were submitted during the peer review process. They are not the versions of record or the final published versions. They should not be cited or distributed as the published version of this manuscript.

BMJ Open is an open access journal and the full, final, typeset and author-corrected version of record of the manuscript is available on our site with no access controls, subscription charges or pay-per-view fees (http://bmjopen.bmj.com).

If you have any questions on BMJ Open's open peer review process please email info.bmjopen@bmj.com

BMJ Open

Linkage of Voluntary Medical Male Circumcision Clients to Adolescent Sexual and Reproductive Health (ASRH) services through Smart-LyncAges project in Zimbabwe: A cohort study

Journal:	*BMJ Open*
Manuscript ID	bmjopen-2019-033035
Article Type:	Research
Date Submitted by the Author:	17-Jul-2019
Complete List of Authors:	Makoni, Talent; Ministry of Health and Child Care, AIDS and TB Unit ; World Health Organization Regional Office for Africa<br>Thekkur, Pruthu; International Union Against Tuberculosis and Lung Disease, Centre for Operational Research; The Union South-East Asia Office, Centre for Operational Research<br>Takarinda, Kudakwashe ; Ministry of Health and Child Care<br>Xaba, Sinokuthemba; Ministry of Health and Child Care<br>Ncube , Getrude ; Ministry of Health and Child Care<br>Zwangobani, Nonhlahla; Ministry of Health and Child Care<br>Samuelson, Julia; World Health Organization<br>Mangombe, Aveneni; Ministry of Health and Child Care<br>Mabaya, Simbarashe; World Health Organization Regional Office for Africa<br>Tapera, Talent ; Ministry of Health and Child Care<br>Matambo, Ronald ; The Union Zimbabwe<br>Ameyan, Wole ; World Health Organization<br>Mugurungi, Owen; Ministry of Health and Child Care
Keywords:	HIV & AIDS < INFECTIOUS DISEASES, Adolescent Health, SORT IT, Operational Research, Sexual and Reproductive Health, Youth centres

SCHOLARONE™
Manuscripts

I, the Submitting Author has the right to grant and does grant on behalf of all authors of the Work (as defined in the below author licence), an exclusive licence and/or a non-exclusive licence for contributions from authors who are: i) UK Crown employees; ii) where BMJ has agreed a CC-BY licence shall apply, and/or iii) in accordance with the terms applicable for US Federal Government officers or employees acting as part of their official duties; on a worldwide, perpetual, irrevocable, royalty-free basis to BMJ Publishing Group Ltd ("BMJ") its licensees and where the relevant Journal is co-owned by BMJ to the co-owners of the Journal, to publish the Work in this journal and any other BMJ products and to exploit all rights, as set out in our licence.

The Submitting Author accepts and understands that any supply made under these terms is made by BMJ to the Submitting Author unless you are acting as an employee on behalf of your employer or a postgraduate student of an affiliated institution which is paying any applicable article publishing charge ("APC") for Open Access articles. Where the Submitting Author wishes to make the Work available on an Open Access basis (and intends to pay the relevant APC), the terms of reuse of such Open Access shall be governed by a Creative Commons licence – details of these licences and which Creative Commons licence will apply to this Work are set out in our licence referred to above.

Other than as permitted in any relevant BMJ Author's Self Archiving Policies, I confirm this Work has not been accepted for publication elsewhere, is not being considered for publication elsewhere and does not duplicate material already published. I confirm all authors consent to publication of this Work and authorise the granting of this licence.

For peer review only

Linkage of Voluntary Medical Male Circumcision Clients to Adolescent Sexual and Reproductive Health (ASRH) services through Smart-LyncAges project in Zimbabwe: A cohort study

Manuscript Type: Research Article

Short Running Title: Linkage of VMMC clients to ASRH services in Zimbabwe

Investigators and affiliations

Talent M. Makoni[1], Pruthu Thekkur[2,3], Kudakwashe C. Takarinda[4,5], Sinokuthemba Xaba[5], Getrude Ncube[6], Nonhlahla Zwangobani[7], Julia Sumuelson[8], Aveneni Mangombe[9], Simbarashe Mabaya[10], Talent Tapera[11], Ronnie Matambo[12], Wole Ameyan[13], Owen Mugurungi[14]

1. National Voluntary Medical Male Circumcision and Adolescent Sexual Reproductive Health Linkages Officer, Ministry of Health, Harare, Zimbabwe

2. Monitoring and Evaluation Officer, Centre for Operational Research, International Union Against Tuberculosis and Lung Diseases, Paris, France

3. The Union-South East Asia Office, New Delhi, India

4. Senior Operational Research Fellow, Centre for Operational Research, The Union, Zimbabwe

5. National Voluntary Medical Male Circumcision Co-ordinator, Ministry of Health, Harare, Zimbabwe

6. National HIV Prevention Co-ordinator, Ministry of Health, Harare, Zimbabwe

For peer review only

7 Director, Technical Services, Zimbabwe National Family Planning Council, Ministry of Health, Harare, Zimbabwe

8. Nurse Epidemiologist, World Health Organization, Geneva, Switzerland

9. National Adolescent Sexual Reproductive Health Co-ordinator, Ministry of Health, Harare, Zimbabwe

10. National Professional Officer, World Health Organization, Zimbabwe

11. Senior Monitoring and Evaluation Officer, Zvandiri Project, AFRICAID, Zimbabwe

12. Operations Officer, The International Union Against Tuberculosis and Lung Diseases, Zimbabwe

13. Technical Officer, Adolescent HIV, World Health Organization, Geneva

14. Director, HIV and TB Programme, Ministry of Health, Harare, Zimbabwe

Corresponding Author:

Talent M. Makoni,

National Voluntary Medical Male Circumcision and Adolescent Sexual Reproductive Health Linkages Officer,

Ministry of Health, Harare, Zimbabwe

Email ID: talent.makoni32@gmail.com

Manuscript details

Word count of abstract - 232; Word count of manuscript - 3408; Tables - 4; Figures - 1; References – 24

For peer review only

ABSTRACT

Objectives: The Smart-LyncAges project was piloted in Bulawayo city and Mt Darwin district of Zimbabwe, to increase utilization of Adolescent Sexual Reproductive Health (ASRH) services through referral of Voluntary Medical Male Circumcision (VMMC) clients to youth centres. Since its inception in 2016, there has been no assessment of performance of the referral system. Thus, this study aimed to assess the proportion of VMMC clients getting 'successfully linked' to ASRH services and factors associated with not being linked.

Design: This was a cohort study using routinely collected secondary data.

Setting: All three VMMC clinics of Mt Darwin district and Bulawayo metropolitan city.

Primary outcome measures: The proportion of 'successfully linked' was summarized as percentage with 95% confidence interval (CI). Adjusted relative risks (aRR) using generalized linear model was calculated as a measure of association between client characteristics and not being linked.

Results: Of the 1478 VMMC clients referred, 463 (31.3%, 95% CI- 30.0-33.8) were 'successfully linked' to ASRH services and the median (IQR) duration for linkage was 6 (0-56) days. Receiving referral from Bulawayo circumcision clinic (RR-1.4 (95% CI-1.2-1.7)) and undergoing circumcision at outreach sites (RR-1.2 (95% CI-1.1-1.3)) were independently associated with not registering for ASRH services.

Conclusion: Linkage to ASRH services from VMMC is feasible as one-third referred of VMMC clients were successfully linked. However, there is need to explore reasons for not accessing ASRH services and take corrective actions to improve the linkages.

Keywords: HIV prevention; Adolescent Health; Sexual and Reproductive Health; Youth centres; SORT IT; Operational Research

For peer review only

Article Summary:

Strengths and Limitations:

- First study to have assessed the extent of linkages with Smart-LyncAges project
- No selection bias as we included all the young people (10-24 years) who registered for circumcision services in the VMMC clinics of two project districts
- Routine programmatic data of large cohort was used and thus reflected the field realities in project implementation
- There might have been an underestimation in proportion of young people ‘successfully linked’ to ASRH services as only those who produced referral slip from VMMC clinic were considered as ‘successfully linked’
- Study was conducted only during two months of the year and thus might have failed to account for seasonal variations in seeking ASRH services

For peer review only

INTRODUCTION

Young people (aged 10-24 years) are disproportionately affected by Human Immunodeficiency Virus (HIV) infection with an estimated 3.9 million living with HIV worldwide in 2017.(1,2) Globally, approximately 1600 youths acquire HIV every day and a youth dies every 10 minutes because of AIDS related illness.(1) In contrast to decline in HIV related death rates in other age groups during 2000 to 2015, there has been a rise among young people.(3) In young people, HIV is the second leading cause of death globally and is the leading cause of death in Africa.(4) In 2017, of all the new adult HIV infections globally, about 16% were among adolescents (15-19 years).(5) About 84% of young people living with HIV are in Sub-Saharan Africa and it is estimated that an additional 7.4 million young people might become infected with HIV in Sub-Saharan Africa region alone by 2030.(6)

Unprotected sex is the most common route of HIV infection among young people and is largely due to low knowledge on HIV and how it can be prevented, early sexual debut, multiple sexual partners, and low condom use.(7) This highlights the need for comprehensive sexuality education (CSE) for adolescents before they become sexually active.[8] In 2001, the United Nations General Assembly Special Session (UNGASS) on HIV set a goal of providing 95% of young people access to information, youth-specific HIV education and life skills development to reduce their vulnerability to HIV infection.(8) Following this, most Sub-Saharan African countries implemented Adolescent Sexual and Reproductive Health (ASRH) services. However, recent studies from various countries in the region have reported poor utilization of ASRH services with only about 21%-51% of adolescents accessing it.[9,10] Acknowledging the importance and poor utilisation of ASRH services, the World Health Organization (WHO) recommends the strengthening of the

For peer review only

linkages between various HIV prevention and ASRH programmes and services and also to integrate the ASRH services with general health services.(11)

Zimbabwe, a landlocked Sub-Saharan country has a high burden of HIV with an estimated prevalence of 14% among people aged 15 to 49 years.(12) The country has ASRH indicators showing risky behaviours with about 40% girls and 30% boys having sex before age eighteen and 62% of males aged 15-24 not using a condom in their last sexual encounter.(13) HIV prevalence in individuals aged 15–19 years was 3.7% and 4.6% for boys and girls respectively. Among those 20-24 years the HIV prevalence was 8% and 10.8% for boys and girls respectively.(13,14) The age-disparity in HIV prevalence among young people, high rate of early sexual debut and low condom use indicates gaps in HIV prevention among adolescents. Nevertheless, since 2009, Zimbabwe has implemented several programmes to limit HIV infection among young people with the two core strategies of Voluntary Medical Male Circumcision (VMMC) and ASRH services.

The ASRH programme implemented in 2010, involves the provision of CSE, life skills training, diagnosis and treatment of Sexually Transmitted Infections (STIs) including HIV, family planning counselling, positive masculinity education, vocational training, library services, recreational games and empowerment on rights and responsibility.(15) The ASRH services are delivered at youth friendly clinics or youth centres. There is anecdotal evidence that the majority of young people do not access ASRH services. Whereas, the VMMC programme, implemented as part of combination HIV prevention strategy as per World Health Organisation (WHO) and the United Nations Joint Programme on HIV/AIDS (UNAIDS) recommendation has a high utilization rate.(16) By 2017, about 80% young boys were covered under VMMC and have had unprecedented contact with the health system.

For peer review only

However, the potential of VMMC services as a gateway to additional relevant ASRH services for young people has not been maximized.

The ASRH and VMMC programs have been implemented as two different vertical national health programs, not complementing each other in improving the service utilization. In 2016, the WHO supported the Ministry of Health and Child Care to pilot Smart-LyncAges project to identify approaches to sustain VMMC and improve the complementarity of both services. Through the project, cross-referral mechanisms were established wherein adolescent boys from VMMC are linked to ASRH programmes and vice versa. Though the Smart-LyncAges project has been piloted as a participatory learning approach since 2016, there was limited quantitative information on the extent of successful linkage of clients between VMMC clinics and ASRH services. This information on extent of successful referral and associated factors was necessary to address any deficiencies in the referral system prior to scale up of the project countrywide. In this regard, the study aimed to assess the proportion of young people (10-24 years) referred from VMMC clinics successfully linked for ASRH services and factors associated with 'not getting registered' in the Smart-LyncAges pilot project sites in Zimbabwe.

For peer review only

METHODOLOGY

Study Design:

This was a cohort study using routinely collected secondary data by the VMMC and ASRH programmes.

Study Setting:

General Setting:

Zimbabwe is a land locked country in Sub Saharan Africa. The country is divided into 10 administrative provinces and has a total of 62 districts. According to the Zimbabwe National Statistics Agency, the country had a projected population of 16,4 million in 2018 and 26% of the total population is adolescents and young adults.(17)

Specific Setting:

The study was conducted in the Bulawayo metropolitan province and Mt Darwin district of Mashonaland Central province, where the VMMC-ASRH linkages project was piloted. Bulawayo is Zimbabwe's second largest city and has a population of approximately 653,000.(17) Mt Darwin is one of seven districts in the Mashonaland Central province with the population of about 213,000. In the district, majority of the population reside in the rural areas.

VMMC services and referral to ASRH

The VMMC programme functions under the National AIDS and TB unit with funding support from external sources mainly The U.S. Presidents Emergency Plan for AIDS Relief (PEPFAR) and Bill and Melinda Gates Foundation (BMGF). The VMMC sites were supported by various non-governmental organizations (NGOs) as implementing partners facilitating

For peer review only

service delivery and community mobilization. The Bulawayo Metropolitan province has two VMMC clinics and the Mount Darwin district has one clinic. The circumcision services were provided at static VMMC clinics and also through outreach clinics in the public health facilities.

Demand generation for VMMC services was created through print and mass media advertisements, school health programmes, road shows and music galas. At community level, the adolescents and young adults were counselled and referred to VMMC clinics by incentivized community mobilizers, peer educators or health workers. The adolescents and young adults registered for ASRH services at youth centres were also referred for VMMC. The adolescents were referred to either static VMMC clinics or outreach clinics based on their convenience.

Upon presentation at a VMMC clinic, the clients were registered with unique VMMC ID number. The clerk documented details of clients in the 'client intake form'. During registration, the clerk issued the referral slip to all the clients who were not already registered for ASRH services. The clients were advised to register themselves for ASRH services in their preferred youth centre. The referral slip was created in triplicate; one slip given to the adolescent, one maintained in the referral file at the VMMC clinic and the other slip sent to peer-educator of the service area from where the adolescent has come.

The adolescents registered in the VMMC were offered group education and individual counselling by nurse counsellor. During counselling, importance of registering for ASRH services was emphasized. The nurse counsellors provided HIV testing and pre-operation examination to assess the eligibility for circumcision surgery. Those adolescents with diabetes, keloids and haemophilia were considered as not eligible for surgery. Among those eligible for surgery, either dorsal slit or forceps guided procedure was performed by

For peer review only

either a trained and qualified nurse circumciser or doctor surgeon. Adolescents were advised to make follow-up visits on day-2 and day-7 after the procedure. The adolescents were also advised to visit the clinic in case of any complications. All the details on surgery, follow-up and adverse events were recorded in the 'client intake form'.

ASRH services and registration

The ASRH services were provided at the youth centres. The Bulawayo metropolitan province has seventeen youth centres and the Mount Darwin district has two youth centres. Each youth centre was supposed to be staffed with a youth health advisor (a registered general nurse), youth facilitator/recreational officer (a social worker) and five peer educators.

Upon arrival at the youth centres, adolescents referred from VMMC submit their VMMC-ASRH referral slips. The youth facilitator/recreational officer registers the adolescents for ASRH services and documents the VMMC ID number in VMMC-ASRH linkages register. The services received by the adolescent during their initial visit were also documented in the VMMC-ASRH linkage register. The referral slip received from the adolescent are stored in the referral box maintained at the youth centre.

Study Population:

All adolescents and young adults (10-24 years) registered in VMMC clinics of Mt Darwin district and Bulawayo city during October and November, 2018 were included in the study. Those who were already registered for ASRH services prior to accessing VMMC services were excluded.

For peer review only

Data Variables, sources of data and data collection:

We extracted details including VMMC identification number, age, education status, mode of referral to VMMC clinic, type of VMMC clinic, HIV status, date of referral to ASRH services, date of registration at VMMC, reason for circumcision, eligibility for circumcision, circumcision status, method of circumcision, status of day-2 post op visit, status of day-7 post op visit and adverse events following circumcision within 42 day from the VMMC 'client intake form' maintained at VMMC clinic. The details on registration and date of registration for ASRH services were extracted from VMMC-ARSH linkage register maintained at youth centres. The information on registration for ASRH services extracted from VMMC-ASRH register was validated using the referral slips maintained in the referral box of youth centres. Those individuals registered for ASRH services within three months of receiving referral slip were considered as 'successfully linked' to ASRH services.

The data was extracted in March, 2019 using two separate structured data extraction proformas designed to extract data from VMMC 'client intake form' and VMMC-ARSH linkage register**.** Data extraction proformas were field tested by the principal investigator and modified before data extraction.

Data entry and analysis:

Data was double entered and validated using EpiData entry software (EpiData Association, Odense, Denmark). Two separate data entry structures were used to enter data from two proformas. The two data structures were then merged using unique 'VMMC ID number'. The final merged data file was used for analysis.

Data was analyzed using Stata version 12.0 (STATA Corp., College, TX, USA). Socio-demographic, HIV status and clinical characteristics was summarized using percentages.

For peer review only

The proportion of participants ‘successfully linked’ to ASRH was summarized as percentages with 95% confidence interval.

The association between socio-demographic and baseline clinical characteristics with ‘not linked for ASRH service’ was assessed using bivariate log binomial regression. To assess the independent association, a generalized linear model (Poisson regression) with variables having p value <0.25 in the bivariate log binomial regression was developed. Adjusted relative risks with 95% CI were calculated as measure of association.

Ethics Approval:

The ethics approval was obtained from the Medical Research Council of Zimbabwe (MRCZ/E229) and Ethics Advisory Group of the International Union Against Tuberculosis and Lung Disease, Paris, France (58/18). Permission was sought from the Ministry of Health and Child Care program officials for extracting the routine data collected at VMMC and ASRH centres.

A data sharing statement:

Technical appendix, statistical code, and dataset available from the https://www.dropbox.com/sh/cm03olkw3qj8j4x/AAArNAzjweJ1iw0UHq86JIFwa?dl=0

Patient and Public Involvement:

Principal Investigator and the data collectors did not interact directly with the young people availing services from VMMC clinics during this retrospective record review. The PI worked with the healthcare staff and peer educators of both the VMMC and ASRH clinics included in the study. Findings from this study will help the Smart-LyncAges project to

For peer review only

assess the performance in linkages and also gives insight on deficiencies to be fixed prior to country wide scale-up.

For peer review only

RESULTS

In total 1773 young people (10-24 years) were registered for VMMC services during the study reference period. Of the total, 1478 (83%) had not been previously registered in ASRH clinic and were included in the study. The mean (SD) age of study participants was 13.7 (4.3) years and 427 (28.9%) were out of school. Of the 1478 study participants, 1032 (69.8%) were referred for VMMC by community mobilizer and 1230 (83.2%) reported HIV prevention as the reason for seeking circumcision service. Of the total, 6 (0.3%) had HIV infection. The characteristics of study participants are depicted in **Table-1**.

Of the 1478 study participants, 1461 (99%) were eligible for circumcision surgery and among of those eligible, 1443 (99%) underwent circumcision (**Figure-1**). Of those who underwent circumcision, 477 (33.1%) had the surgery performed at an outreach site. Of the 1443 participants, 1379 (95.6%) and 1025 (71%) attended day 2 and day 7 review visits respectively. In total, 20 (1.4%) developed mild adverse event following circumcision and 3 (0.2%) each developed moderate and severe adverse events (**Table-2**).

Of the 1478 study participants referred for ASRH services, 463 (31.3%, 95% CI-30.0%-33.8%) successfully linked for ASRH services at ‘youth centre’ within 3 months of referral (**Figure-1**). Among those who were successfully linked, the median (IQR) duration from referral to getting registered at ‘youth centre’ was 6 (0-56) days. Referral from Bulawayo circumcision clinic (RR-1.4 (95% CI-1.2-1.7)) and undergoing circumcision at outreach sites (RR-1.2 (95% CI-1.1-1.3)) were independently associated with not linked for ASRH services after referral from circumcision clinic. The association between participant’s characteristics and failure of referral is shown in **Table-3**.

For peer review only

DISCUSSION

This is the first study assessing the extent of successful referral among adolescents and young men between VMMC clinics and ASRH services under Smart-LyncAges project implemented in Zimbabwe. About 31% of young males (10-24 years) referred from VMMC clinics successfully linked to ASRH services at youth centres within three months of referral. Young people referred from the outreach sites and Bulawayo male circumcision clinic had significantly higher rates of not being registered for ASRH services.

Globally, there were limited studies assessing the linkages between various HIV preventive strategies like VMMC services and ASRH services. A study from Zambia reported that, with enhanced counselling and referral from community, uptake of ASRH services like HIV testing and counselling, family planning counselling and VMMC services was less than 20% within six months.(18) Also, previous reviews have reported that demand generation activities in the community, improving accessibility and quality of ASRH services improve ASRH related knowledge but may not increase the utilization of specific ASRH services.(19–21) The potential reason for underutilization of ASRH services may be that, a preventive strategy may not be considered as an immediate need by young people.(21–23) Considering this, the successful linkage (uptake) of about one in three of the VMMC clients to ASRH services through Smart-LyncAges project is promising. It would be important to better understand the real and perceived ASRH needs and the relevance of referrals.

The clients referred from Bulawayo MC clinic had slightly higher risk of not being linked to ASRH services. A potential reason for this finding is that, the MC clinic is situated in the eastern suburbs of the city where people of high socio-economic status reside. All the fifteen youth centres are however were located in western suburbs which are predominantly of low socio economic status. It is possible that, the recreational activities

For peer review only

317 available under ASRH services do not appeal to the technologically savvy urban youths from
318 the elite suburbs. Furthermore, distance to youth centres might have limited the clients
319 from the eastern suburbs to register for ASRH services. Similarly, clients accessing VMMC
320 services at outreach clinics were not being linked to ASRH services. The focus of outreach
321 clinics is to conduct surgery in hard to reach areas. Though the referral slips were issued,
322 there would not be space and time to counsel on ASRH services comprehensively. Also, the
323 youth centres in Mt Darwin were mainly located in district centres and might not be
324 accessible to clients of outreach clinics. Although the actual numbers were small, a high
325 proportion of clients ineligible for circumcision or experiencing an adverse event were
326 referred and successfully linked suggesting they have additional service needs.

327 The study had several strengths. First, the study was conducted within the routine
328 programmatic setting, reflecting the field realities and using routine data. Second, all the
329 young males attending the VMMC clinics during the study reference period were included
330 and thus, there was no sampling bias. Third, the study had large sample size and thus, the
331 estimate of 'successfully linked' was precise. Fourth, the continuous supportive supervision
332 was rendered during the study period to ensure completeness in recording under project
333 setting and thus, the 'missing data' was limited. Fifth, the registration for ASRH services was
334 validated by cross-checking the stored referral slips along with information extracted from
335 VMMC-ASRH linkage register and thus, it enhanced the validity of the outcome
336 ascertainment. Sixth, the STrengthening the Reporting of OBservational Studies in
337 Epidemiology (STROBE) guidelines was used to report the study findings.(24)

338 The study had a few limitations. First, the registration for ASRH services was
339 ascertained based on the VMMC-ASRH linkage register. Only, those who produced VMMC
340 referral slips during registration were listed in VMMC-ASRH register. Thus, those

For peer review only

participants who might have missed their referral slips before registration for ASRH services could have been misclassified as 'not linked'. This could have led to underestimation of percentage of 'successfully' linked. Second, the study was conducted only during two months of the year and thus failed to account for seasonal variations in seeking services. During the festive month of December, the youth centres were closed due to holidays. Also, during December, the youth centres in the Mt Darwin region were closed for two weeks due to cholera outbreak in the region. There might have been an underestimation of 'successfully' linked due to non-functioning of youth centres during study follow-up period. Third, the pathways for seeking ASRH services and contribution of peer-educators in reaching the ASRH services were not assessed. Thus, the study failed to document the pathways with highest successful linkage rate, which could have helped in replicating the model elsewhere. Fourth, the potential confounders like distance from youth centres, socio-economic status, parents' willingness and adolescents' willingness were not captured and included in the adjusted analysis. Inclusion of these variables could have improved the validity of the model. Fifth, because the study represents select group of young people who came for VMMC clinics in the project pilot districts, thus the generalizability of the study results is limited. Lastly, study did not explore the reasons for not registering for ASRH services among those referred.

The study has a few programmatic implications and recommendations. First, the percentage of 'successfully linked' between VMMC clinics and ASRH services was promising. The Smart-LyncAges project can be scaled up to improve the utilization of ASRH services. However, there is need for qualitative research to explore the reasons for not accessing the ASRH services among those who did not register. This information can help to improve

For peer review only

linkages and effectiveness by understanding relevance of linkages to young people`s health needs.

Second, with the existing records maintained under the project, cohort monitoring of the performance of referral system was feasible. The ‘proportion of those referred from the facility successfully registered for ASRH services’ can be introduced as an indicator in the monthly report of each VMMC clinic. This would enable the programme managers to monitor the performance of referral system better than the absolute number of referred which is being reported now.

Third, the young adolescents seeking VMMC services from outreach clinics at peripheral public health facilities were not registering for ASRH services, implying the distance to youth centres was a potential barrier. Solutions should be person centred, such as co-location of ASRH and VMMC services, improved accessibility with services decentralized to peripheral public health facilities and enhanced training of existing general health staff to deliver youth friendly services.

Fourth, about 70% of clients came back to avail follow-up services on day-7 post circumcision. This opportunity can be used to further educate and to reinforce to young people on services available to them as they will be more able to absorb information since the MC procedure has been completed.

CONCLUSION

The rate of young males referred from VMMC clinics and successfully linked for ASRH services was promising. However, there is need to explore the reasons for clients not accessing the ASRH services, the type of services sought and take corrective actions to improve the same.

For peer review only

ACKNOWLEDGEMENTS

This research was conducted through the Structured Operational Research and Training Initiative (SORT IT), a global partnership led by the Special Programme for Research and Training in Tropical Diseases at the World Health Organization (WHO/TDR). The training model is based on a course developed jointly by the International Union Against Tuberculosis and Lung Disease (The Union) and Medécins sans Frontières (MSF). The specific SORT IT program which resulted in this publication was implemented by the Centre for Operational Research, The Union, Paris, France. Mentorship and the coordination/facilitation of this particular SORT IT workshop was provided through the Centre for Operational Research, The Union, Paris, France; the Department of Tuberculosis and HIV, The Union, Paris, France; The Union, Zimbabwe Office; The Union, South East Asia Office; University of Washington, School of Public Health, Department of Global Health, Seattle, Washington, USA; National Institute for Medical Research, Muhimbili Centre, Dar es Salaam, Tanzania; and AIDS & TB Department, Ministry of Health & Child Care, Harare, Zimbabwe

FUNDING

The training course under which this study was conducted was funded by: the United Kingdom's Department for International Development (DFID); The Global Fund to Fight AIDS, Tuberculosis and Malaria (GFATM) and the World Health Organization (WHO). The WHO also provided funding support for conduct of the study. The funders had no role in study design, data collection and analysis, decision to publish, or preparation of the manuscript.

For peer review only

AUTHOR CONTRIBUTION

TM was the principal investigator; PT and KT were the SORT IT course mentors; OM, WA, JS, SX and GN were the senior authors. TM, NZ, AM, SM, TT, and RM were involved in data collection; TM, PT and KT analyzed the data and prepared the first draft of the paper. All authors were involved in conception, design, inference of results, providing critical review and approval of the final manuscript.

DISCLOSURE STATEMENT

None of the authors have any competing interests.

For peer review only

REFERENCES

1. Joint United Nations Programme on HIV/AIDS (UNAIDS). Youth and HIV — Mainstreaming a three-lens approach to youth participation [Internet]. Geneva, Switzerland; 2018 [cited 2019 Jun 10]. Available from: https://www.unaids.org/sites/default/files/media_asset/youth-and-hiv_en.pdf
2. Joint United Nations Programme on HIV/AIDS (UNAIDS). Start Free, Stay Free, AIDS Free [Internet]. Geneva, Switzerland; 2015 [cited 2019 May 8]. Available from: http://www.unaids.org/sites/default/files/media_asset/JC2869_Be Free Booklet_A4.pdf
3. UNICEF. Adolescent deaths from AIDS tripled since 2000 – UNICEF | Press centre | UNICEF [Internet]. UNICEF. [cited 2019 May 8]. Available from: https://www.unicef.org/media/media_86384.html
4. World Health Organization (WHO). WHO | Adolescent deaths and burden of disease [Internet]. WHO. World Health Organization; 2017 [cited 2019 May 8]. Available from: https://www.who.int/maternal_child_adolescent/epidemiology/adolescent-deaths-burden-disease/en/
5. Joint United Nations Programme on HIV/AIDS (UNAIDS). Global HIV & AIDS statistics — 2018 fact sheet [Internet]. Geneva, Switzerland; 2018 [cited 2019 May 8]. Available from: http://www.unaids.org/sites/default/files/media_asset/UNAIDS_FactSheet_en.pdf
6. UNICEF. END AIDS Seventh Stocktaking Report, 2016 [Internet]. New York, USA; 2016 [cited 2019 May 8]. 1-92 p. Available from: www.childrenandaids.org
7. Joint United Nations Programme on HIV/AIDS (UNAIDS). HIV prevention among adolescent girls and young women [Internet]. 2016 [cited 2019 May 8]. Available

For peer review only

from:
http://www.unaids.org/sites/default/files/media_asset/UNAIDS_HIV_prevention_among_adolescent_girls_and_young_women.pdf

8. United Nations General Assembly Special Session on HIV/AIDS. Summary of the Declaration of Commitment on HIV/AIDS - United Nations General Assembly, Special Session on HIV/AIDS, 25-27 June 2001, New York [Internet]. New York, USA; 2001 [cited 2019 May 8]. Available from: http://www.unaids.org

9. Binu W, Marama T, Gerbaba M, Sinaga M. Sexual and reproductive health services utilization and associated factors among secondary school students in Nekemte town, Ethiopia. [cited 2019 May 9]; Available from: https://doi.org/10.1186/s12978-018-0501-z

10. Feleke SA, Koye DN, Demssie AF, Mengesha ZB. Reproductive health service utilization and associated factors among adolescents (15–19 years old) in Gondar town, Northwest Ethiopia. BMC Health Services Research [Internet]. 2013 Dec 3 [cited 2019 May 9];13(1):294. Available from: https://bmchealthservres.biomedcentral.com/articles/10.1186/1472-6963-13-294

11. The importance of sexual and reproductive health and rights to prevent HIV in adolescent girls and young women in eastern and southern Africa Evidence brief [Internet]. 2017 [cited 2019 May 9]. Available from: https://apps.who.int/iris/bitstream/handle/10665/255334/WHO-RHR-17.05-eng.pdf;jsessionid=4E61673B4AD0E4428DF9AEDC899B5131?sequence=1

12. Ministry of Health and Child Care. ZIMBABWE POPULATION-BASED HIV IMPACT ASSESSMENT ZIMPHIA 2015-16 [Internet]. Harare; 2016 [cited 2019 May 9]. Available from: https://phia.icap.columbia.edu/wp-content/uploads/2016/11/ZIMBABWE-

For peer review only

Factsheet.FIN_.pdf

13. Ministry of Health and Child Care. Zimbabwe Demographic and Health Survey [Internet]. Harare, Zimbabwe; 2015 [cited 2019 May 9]. Available from: https://dhsprogram.com/pubs/pdf/FR322/FR322.pdf

14. Zimbabwe National Statistics Agency. The Zimbabwe Multiple Indicator Cluster [Internet]. Harae; 2015 [cited 2019 May 9]. Available from: http://www.zimstat.co.zw/sites/default/files/img/MICS_REPORT_2014.pdf

15. Ministry of Health and Child Welfare. National Adolescent Sexual and Reproductive Health Strategy, 2010–2015. Harare, Zimbabwe; 2009.

16. Ministry of Health and Child Care. Accelerated Strategic and Operational Plan 2014-2018. Harare, Zimbabwe; 2019.

17. Zimbabwe National Statistics Agency. ZIMBABWE POPULATION CENSUS 2012 [Internet]. Harare, Zimbabwe; 2012 [cited 2019 May 9]. Available from: http://www.zimstat.co.zw/sites/default/files/img/publications/Population/National_Report.pdf

18. Hewett PC, Nalubamba M, Bozzani F, Digitale J, Vu L, Yam E, et al. Randomized evaluation and cost-effectiveness of HIV and sexual and reproductive health service referral and linkage models in Zambia. BMC Public Health [Internet]. 2016 Dec 12 [cited 2019 May 9];16(1):785. Available from: http://bmcpublichealth.biomedcentral.com/articles/10.1186/s12889-016-3450-x

19. Mazur A, Brindis CD, Decker MJ. Assessing youth-friendly sexual and reproductive health services: a systematic review. BMC Health Services Research [Internet]. 2018 Dec 27 [cited 2019 May 9];18(1):216. Available from: https://bmchealthservres.biomedcentral.com/articles/10.1186/s12913-018-2982-4

For peer review only

494 20. Denno DM, Hoopes AJ, Chandra-Mouli V. Effective Strategies to Provide Adolescent
495 Sexual and Reproductive Health Services and to Increase Demand and Community
496 Support. Journal of Adolescent Health [Internet]. 2015 Jan 1 [cited 2019 May
497 9];56(1):S22–41. Available from:
498 https://www.sciencedirect.com/science/article/pii/S1054139X14004248
499 21. Chandra-Mouli V, Lane C, Wong S. What Does Not Work in Adolescent Sexual and
500 Reproductive Health: A Review of Evidence on Interventions Commonly Accepted as
501 Best Practices. Global health, science and practice [Internet]. 2015 Aug 31 [cited 2019
502 May 9];3(3):333–40. Available from:
503 http://www.ncbi.nlm.nih.gov/pubmed/26374795
504 22. Cassidy C, Bishop A, Steenbeek A, Langille D, Martin-Misener R, Curran J. Barriers and
505 enablers to sexual health service use among university students: a qualitative
506 descriptive study using the Theoretical Domains Framework and COM-B model. BMC
507 Health Services Research [Internet]. 2018 Dec 24 [cited 2019 May 9];18(1):581.
508 Available from:
509 https://bmchealthservres.biomedcentral.com/articles/10.1186/s12913-018-3379-0
510 23. Travers M, O'Uhuru D, Mueller T, Bedell J. Implementing Adolescent Sexual and
511 Reproductive Health Clinical Best Practice in the Bronx, New York. Journal of
512 Adolescent Health [Internet]. 2019 Mar [cited 2019 May 9];64(3):376–81. Available
513 from: https://linkinghub.elsevier.com/retrieve/pii/S1054139X1830435X
514 24. von Elm E, Altman DG, Egger M, Pocock SJ, Gøtzsche PC, Vandenbroucke JP, et al. The
515 Strengthening the Reporting of Observational Studies in Epidemiology (STROBE)
516 statement: guidelines for reporting observational studies. The Lancet [Internet]. 2007
517 Oct 20 [cited 2019 Jun 10];370(9596):1453–7. Available from:

For peer review only

http://www.ncbi.nlm.nih.gov/pubmed/18064739

For peer review only

Table 1: The demographic characteristics, reason for circumcision and HIV status of adolescents and young adults registered for VMMC services during October and November, 2018 in selected health facilities of Zimbabwe, N=1478

Characteristics	Categories	Frequency	(%)
**Total**		**1478**	**(100)**
**Age (in years)**	10-14	980	(66.3)
	15-19	314	(21.2)
	20-24	184	(12.3)
**Education status**	Out of school	427	(28.9)
	Primary education	899	(60.8)
	Secondary education	152	(10.3)
**Health facility**	Mt Darwin	542	(36.7)
	Lobengula MC clinic	587	(39.7)
	Bulawayo MC clinic	349	(23.6)
**Referred by**	Friend or partner	37	(2.5)
	Health worker	216	(14.6)
	Community mobilizer	1032	(69.8)
	Others	139	(9.4)
	Missing	54	(3.7)
**Reasons for circumcision***	HIV prevention	1230	(83.2)
	Sexual pleasure	248	(16.8)
	STI Prevention	736	(49.8)
	Hygiene	1196	(80.9)
	Socio-cultural reasons	51	(3.5)
**HIV status**	Positive	6	(0.3)
	Negative	1472	(99.7)

Abbreviation: HIV- Human Immunodeficiency virus; VMMC- Voluntary Medical Male Circumcision; MC- Male Circumcision; STI- Sexually Transmitted Infection

** Multiple responses are possible*

For peer review only

Table 2: The surgical and follow-up details of adolescents and young adults who underwent circumcision during October and November, 2018 in selected male circumcision clinics of Zimbabwe, N=1443

Characteristics	Categories	Frequency	(%)
**Total**		**1443**	**(100.0)**
**Method of circumcision**	Dorsal slit	1439	(99.7)
	Forceps Guided	4	(0.3)
**Service delivery point**	Static site	966	(66.9)
	Outreach site	477	(33.1)
**Circumciser**	Nurse practitioner	1418	(98.3)
	Doctor	25	(1.7)
**Attended post-op day-2**	Yes	1379	(95.6)
	No	64	(4.4)
**Attended post-op day-7**	Yes	1025	(71.0)
	No	418	(29.0)
**Adverse event**	None	1417	(98.2)
	Mild	20	(1.4)
	Moderate	3	(0.2)
	Severe	3	(0.2)

For peer review only

560 Table 3: Demographic, HIV status and surgical characteristics associated with not getting
561 registered for ASRH services within 3 months of referral from the selected VMMC health
562 facilities in Zimbabwe, October and November 2018.

Characteristic	Category	Total	Not linked to ASRH, n (%)*	Unadjusted RR (95%. CI)	Adjusted RR (95 % CI)#	p value
**Total**		**1478**	**1015 (68.7)**			
**Age (in years)**	10 - 14	980	671 (68.5)	1.1 (1.0-1.2)	1.0 (0.9-1.2)	0.887
	15 - 19	314	200 (63.7)	1	1	
	20 - 24	184	144 (78.3)	1.2 (1.1-1.4)	1.1 (1.0-1.3)	0.149
**Education**	Out of school	427	297 (69.6)	1.1 (1.0-1.3)	1.0 (0.9-1.2)	0.710
	Primary	899	622 (69.2)	1.1 (1.0-1.2)	1.0 (0.9-1.2)	0.713
	Secondary	152	96 (63.2)	1	1	
**Referred By**	Friend/Partner	37	22 (59.5)	1	1	
	Health Worker	216	164 (75.9)	1.3 (1.0-1.7)	1.2 (0.9-1.5)	0.281
	Community mobiliser	1032	694 (67.3)	1.1 (0.9-1.5)	1.1 (0.8-1.4)	0.569
	Others	139	106 (76.3)	1.3 (1.0-1.7)	1.0 (0.8-1.3)	0.973
	Missing	54	29 (53.7)	0.9 (0.6-1.3)	1.1 (0.8-1.6)	0.446
**Health Facility**	Mt Darwin	542	330 (60.9)	1	1	
	Lobengula MC	587	386 (65.8)	1.1 (1.0-1.2)	1.1 (1.0-1.3)	0.118
	Bulawayo MC	349	299 (85.7)	1.4 (1.3-1.5)	1.4 (1.2-1.7)	<0.001
**HIV Status**	Positive	6	5 (83.3)	1.2 (0.8-1.7)	1.3 (0.9-1.9)	0.118
	Negative	1472	1010 (68.6)	1	1	
**Reasons for MC**						
HIV Prevention	Yes	1230	865 (85.2)	1.2 (1.0-1.3)	1.0 (0.9-1.1)	0.815
	No	248	150 (60.5)	1	1	
Sexual Pleasure	Yes	248	158 (63.7)	0.9 (0.8-1.0)	0.9 (0.8-1.1)	0.550
	No	1230	857 (69.7)	1	1	
STI Prevention	Yes	736	523 (71.1)	1.1 (1.0-1.1)	1.0 (0.9-1.1)	0.990
	No	742	492 (66.3)	1	1	
Hygiene	Yes	1196	847(70.8)	1.2 (1.1-1.3)	1.1 (1.0-1.2)	0.192
	No	282	168 (59.6)	1	1	
Socio-cultural	Yes	51	32 (62.8)	0.1 (0.7-1.1)	0.9 (0.7-1.1)	0.432
	No	1427	983 (68.9)	1	1	
**Circumcision¥**	Not Eligible	17	3 (17.7)	1	1	
	Circumcised	1443	1003 (69.5)	3.9 (1.4-11)	1.9 (0.6-6.3)	0.313
	Not circumcised	18	9 (50.0)	2.8 (0.9-8.7)	2.3 (0.8-7.2)	0.139
**Circumciser¥**	Nurse	1418	989 (69.8)	1.2 (0.9-1.8)	1.2 (0.8-1.7)	0.312
	Doctors	25	14 (56.0)	1	1	
**Service delivery point¥**	Static	966	66 (65.8)	1	1	
	Outreach	477	367 (76.9)	1.2 (1.1-1.2)	1.2 (1.1-1.3)	<0.001
**Day-2 follow-up¥**	Yes	1379	965 (70.0)	1.2 (1.0-1.4)	1.1 (0.9-1.4)	0.376
	No	64	38 (59.4)	1	1	
**Day-7 follow-up¥**	Yes	1025	732 (71.4)	1.1 (1.0-1.2)	0.9 (0.7-1.0)	0.042
	No	418	271 (64.8)	1	1	
**Adverse event$¥**	Yes	26	11 (42.3)	1	1	
	No	1417	992 (70.0)	1.7 (1.1-2.6)	1.5 (0.9-2.3)	0.101

For peer review only

Abbreviations: ASRH-Adolescent Sexual Reproductive Health; STI-Sexually Transmitted Infection; HIV-Human Immunodeficiency Virus; RR-Relative Risk, CI-Confidence Interval
* Row percentage; # Using generalized linear model (Poisson regression); $ Mild, Moderate and Severe were clubbed as 'Yes'; ¥ Applicable only for those who underwent circumcision

Figure-1: Flow-chart depicting the adolescents eligible for referral to ASRH services, circumcision status and linkage to ASRH services among those registered at selected VMMC clinics of Zimbabwe during October to November 2018

Abbreviation: VMMC- Voluntary Medical Male Circumcision; ASRH- Adolescent Sexual Reproductive Health

For peer review only

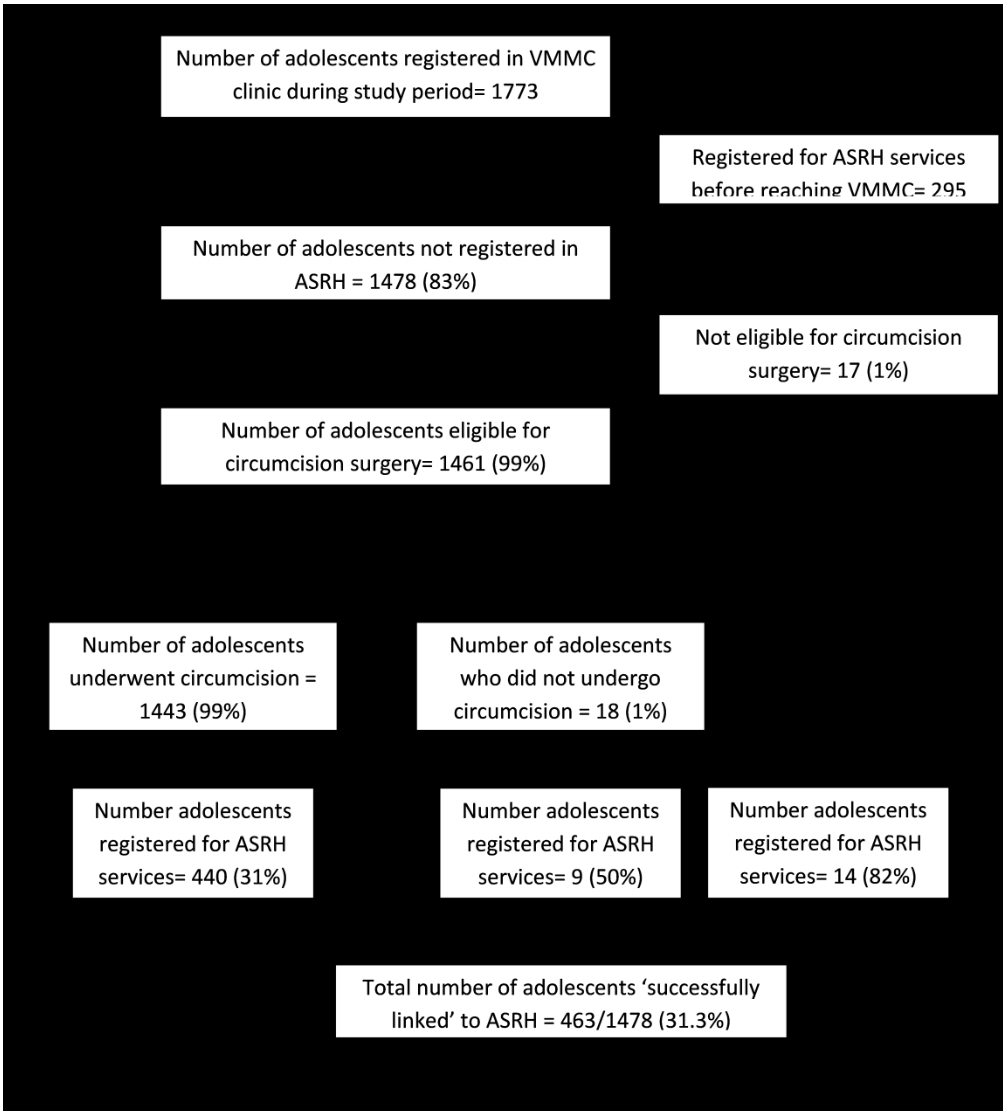

Figure-1: Flow-chart depicting the adolescents eligible for referral to ASRH services, circumcision status and linkage to ASRH services among those registered at selected VMMC clinics of Zimbabwe during October to November 2018

Abbreviation: VMMC- Voluntary Medical Male Circumcision; ASRH- Adolescent Sexual Reproductive Health

359x398mm (300 x 300 DPI)

STROBE Statement

Checklist of items that should be included in reports of observational studies

Section/Topic	Item No	Recommendation	Reported on Page No (Line No)
**Title and abstract**	1	(*a*) Indicate the study's design with a commonly used term in the title or the abstract	1(2-3)
		(*b*) Provide in the abstract an informative and balanced summary of what was done and what was found	3(38-67)
**Introduction**			
Background/rationale	2	Explain the scientific background and rationale for the investigation being reported	5-7(85-145)
Objectives	3	State specific objectives, including any prespecified hypotheses	7(142-145)
**Methods**			
Study design	4	Present key elements of study design early in the paper	8(149-150)
Setting	5	Describe the setting, locations, and relevant dates, including periods of recruitment, exposure, follow-up, and data collection	8-10(153-210)
Participants	6	(*a*) *Cohort study*—Give the eligibility criteria, and the sources and methods of selection of participants. Describe methods of follow-up<br>*Case-control study*—Give the eligibility criteria, and the sources and methods of case ascertainment and control selection. Give the rationale for the choice of cases and controls<br>*Cross-sectional study*—Give the eligibility criteria, and the sources and methods of selection of participants	10(213-216)
		(*b*) *Cohort study*—For matched studies, give matching criteria and number of exposed and unexposed<br>*Case-control study*—For matched studies, give matching criteria and the number of controls per case	
Variables	7	Clearly define all outcomes, exposures, predictors, potential confounders, and effect modifiers. Give diagnostic criteria, if applicable	11(220-230)
Data sources/measurement	8*	For each variable of interest, give sources of data and details of methods of assessment (measurement). Describe comparability of assessment methods if there is more than one group	11(231-234)
Bias	9	Describe any efforts to address potential sources of bias	11(237-240, 245-249)
Study size	10	Explain how the study size was arrived at	10(213-216)
Quantitative variables	11	Explain how quantitative variables were handled in the analyses. If applicable, describe which groupings were chosen and why	11,12(241-244)
Statistical methods	12	(*a*) Describe all statistical methods, including those used to control for confounding	11,12(241-249)
		(*b*) Describe any methods used to examine subgroups and interactions	
		(*c*) Explain how missing data were addressed	11(229-230)

For peer review only - http://bmjopen.bmj.com/site/about/guidelines.xhtml

		(*d*) *Cohort study*—If applicable, explain how loss to follow-up was addressed<br>*Case-control study*—If applicable, explain how matching of cases and controls was addressed<br>*Cross-sectional study*—If applicable, describe analytical methods taking account of sampling strategy	NA
		(*e*) Describe any sensitivity analyses	

Section/Topic	Item No	Recommendation	Reported on Page No(Line No)
**Results**			
Participants	13*	(a) Report numbers of individuals at each stage of study—eg numbers potentially eligible, examined for eligibility, confirmed eligible, included in the study, completing follow-up, and analysed	14(277-280)
		(b) Give reasons for non-participation at each stage	
		(c) Consider use of a flow diagram	Figure-1
Descriptive data	14*	(a) Give characteristics of study participants (eg demographic, clinical, social) and information on exposures and potential confounders	14(270-276)
		(b) Indicate number of participants with missing data for each variable of interest	
		(c) *Cohort study*—Summarise follow-up time (eg, average and total amount)	14(283-286)
Outcome data	15*	*Cohort study*—Report numbers of outcome events or summary measures over time	14(283-284)
		Case-control study—Report numbers in each exposure category, or summary measures of exposure	
		Cross-sectional study—Report numbers of outcome events or summary measures	
Main results	16	(*a*) Give unadjusted estimates and, if applicable, confounder-adjusted estimates and their precision (eg, 95% confidence interval). Make clear which confounders were adjusted for and why they were included	14(286-290)
		(*b*) Report category boundaries when continuous variables were categorized	26 (525)
		(*c*) If relevant, consider translating estimates of relative risk into absolute risk for a meaningful time period	
Other analyses	17	Report other analyses done—eg analyses of subgroups and interactions, and sensitivity analyses	
**Discussion**			
Key results	18	Summarise key results with reference to study objectives	15(294-299)
Limitations	19	Discuss limitations of the study, taking into account sources of potential bias or imprecision. Discuss both direction and magnitude of any potential bias	16,17 (338-356)
Interpretation	20	Give a cautious overall interpretation of results considering objectives, limitations, multiplicity of analyses, results from similar studies, and other relevant evidence	15(300-311)
Generalisability	21	Discuss the generalisability (external validity) of the study results	17(355-357)
**Other Information**			

For peer review only

Funding	22	Give the source of funding and the role of the funders for the present study and, if applicable, for the original study on which the present article is based	19 (407-411)

**Give information separately for cases and controls in case-control studies and, if applicable, for exposed and unexposed groups in cohort and cross-sectional studies.*

Note: An Explanation and Elaboration article discusses each checklist item and gives methodological background and published examples of transparent reporting. The STROBE checklist is best used in conjunction with this article (freely available on the Web sites of PLoS Medicine at http://www.plosmedicine.org/, Annals of Internal Medicine at http://www.annals.org/, and Epidemiology at http://www.epidem.com/). Information on the STROBE Initiative is available at www.strobe-statement.org.

For peer review only

BMJ Open

Linkage of Voluntary Medical Male Circumcision Clients to Adolescent Sexual and Reproductive Health (ASRH) services through Smart-LyncAges project in Zimbabwe: A cohort study

Journal:	*BMJ Open*
Manuscript ID	bmjopen-2019-033035.R1
Article Type:	Original research
Date Submitted by the Author:	02-Oct-2019
Complete List of Authors:	Makoni, Talent; Ministry of Health and Child Care, AIDS and TB Unit ; World Health Organization Regional Office for Africa<br>Thekkur, Pruthu; International Union Against Tuberculosis and Lung Disease, Centre for Operational Research; The Union South-East Asia Office, Centre for Operational Research<br>Takarinda, Kudakwashe ; Ministry of Health and Child Care<br>Xaba, Sinokuthemba; Ministry of Health and Child Care<br>Ncube , Getrude ; Ministry of Health and Child Care<br>Zwangobani, Nonhlahla; Ministry of Health and Child Care<br>Samuelson, Julia; World Health Organization<br>Mangombe, Aveneni; Ministry of Health and Child Care<br>Mabaya, Simbarashe; World Health Organization Regional Office for Africa<br>Tapera, Talent ; Ministry of Health and Child Care<br>Matambo, Ronald ; The Union Zimbabwe<br>Ameyan, Wole ; World Health Organization<br>Mugurungi, Owen; Ministry of Health and Child Care
**Primary Subject Heading**:	Sexual health
Secondary Subject Heading:	Global health, HIV/AIDS, Sexual health, Reproductive medicine, Epidemiology
Keywords:	HIV & AIDS < INFECTIOUS DISEASES, Adolescent Health, SORT IT, Operational Research, Sexual and Reproductive Health, Youth centres

SCHOLARONE™
Manuscripts

I, the Submitting Author has the right to grant and does grant on behalf of all authors of the Work (as defined in the below author licence), an exclusive licence and/or a non-exclusive licence for contributions from authors who are: i) UK Crown employees; ii) where BMJ has agreed a CC-BY licence shall apply, and/or iii) in accordance with the terms applicable for US Federal Government officers or employees acting as part of their official duties; on a worldwide, perpetual, irrevocable, royalty-free basis to BMJ Publishing Group Ltd ("BMJ") its licensees and where the relevant Journal is co-owned by BMJ to the co-owners of the Journal, to publish the Work in this journal and any other BMJ products and to exploit all rights, as set out in our licence.

The Submitting Author accepts and understands that any supply made under these terms is made by BMJ to the Submitting Author unless you are acting as an employee on behalf of your employer or a postgraduate student of an affiliated institution which is paying any applicable article publishing charge ("APC") for Open Access articles. Where the Submitting Author wishes to make the Work available on an Open Access basis (and intends to pay the relevant APC), the terms of reuse of such Open Access shall be governed by a Creative Commons licence – details of these licences and which Creative Commons licence will apply to this Work are set out in our licence referred to above.

Other than as permitted in any relevant BMJ Author's Self Archiving Policies, I confirm this Work has not been accepted for publication elsewhere, is not being considered for publication elsewhere and does not duplicate material already published. I confirm all authors consent to publication of this Work and authorise the granting of this licence.

For peer review only

Linkage of Voluntary Medical Male Circumcision Clients to Adolescent Sexual and Reproductive Health (ASRH) services through Smart-LyncAges project in Zimbabwe: A cohort study

Manuscript Type: Research Article

Short Running Title: Linkage of VMMC clients to ASRH services in Zimbabwe

Investigators and affiliations

Talent M. Makoni[1], Pruthu Thekkur[2,3], Kudakwashe C. Takarinda[4,5], Sinokuthemba Xaba[5], Getrude Ncube[6], Nonhlahla Zwangobani[7], Julia Sumuelson[8], Aveneni Mangombe[9], Simbarashe Mabaya[10], Talent Tapera[11], Ronald Matambo[12], Wole Ameyan[13], Owen Mugurungi[14]

1. National Voluntary Medical Male Circumcision and Adolescent Sexual Reproductive Health Linkages Officer, Ministry of Health, Harare, Zimbabwe

2. Monitoring and Evaluation Officer, Centre for Operational Research, International Union Against Tuberculosis and Lung Diseases, Paris, France

3. The Union-South East Asia Office, New Delhi, India

4. Senior Operational Research Fellow, Centre for Operational Research, The Union, Zimbabwe

5. National Voluntary Medical Male Circumcision Co-ordinator, Ministry of Health, Harare, Zimbabwe

6. National HIV Prevention Co-ordinator, Ministry of Health, Harare, Zimbabwe

For peer review only

7 Director, Technical Services, Zimbabwe National Family Planning Council, Ministry of Health, Harare, Zimbabwe

8. Nurse Epidemiologist, World Health Organization, Geneva, Switzerland

9. National Adolescent Sexual Reproductive Health Co-ordinator, Ministry of Health, Harare, Zimbabwe

10. National Professional Officer, World Health Organization, Zimbabwe

11. Senior Monitoring and Evaluation Officer, Zvandiri Project, AFRICAID, Zimbabwe

12. Operations Officer, The International Union Against Tuberculosis and Lung Diseases, Zimbabwe

13. Technical Officer, Adolescent HIV, World Health Organization, Geneva

14. Director, HIV and TB Programme, Ministry of Health, Harare, Zimbabwe

Corresponding Author:

Talent M. Makoni,

National Voluntary Medical Male Circumcision and Adolescent Sexual Reproductive Health Linkages Officer,

Ministry of Health, Harare, Zimbabwe

Email ID: talent.makoni32@gmail.com

For peer review only

Manuscript details

Word count of abstract - 232; Word count of manuscript - 3408; Tables - 4; Figures - 1; References – 24

ABSTRACT

Objectives: World Health Organization recommended strengthening the linkages between various HIV prevention programmes and Adolescent Sexual Reproductive Health (ASRH) services. The Smart-LyncAges project piloted in Bulawayo city and Mt Darwin district of Zimbabwe, established referral system to link the Voluntary Medical Male Circumcision (VMMC) clients to ASRH services provided at youth centres. Since its inception in 2016, there has been no assessment of the performance of referral system. Thus, we aimed to assess the proportion of young (10-24 years) VMMC clients getting 'successfully linked' to ASRH services and factors associated with not being linked.

Design: This was a cohort study using routinely collected secondary data.

Setting: All three VMMC clinics of Mt Darwin district and Bulawayo metropolitan city.

Primary outcome measures: The proportion of 'successfully linked' was summarized as percentage with 95% confidence interval (CI). Adjusted relative risks (aRR) using generalized linear model was calculated as a measure of association between client characteristics and not being linked.

Results: Of 1773 young people registered for VMMC services during the study reference period, 1478 (83%) who had not previously registered for ASRH services included in the study. The mean (SD) age of study participants was 13.7 (4.3) years and 427 (28.9%) were out of school. Of the 1478 VMMC clients referred, 463 (31.3%, 95% CI- 30.0-33.8) were 'successfully linked' to ASRH services and the median (IQR) duration for linkage was 6 (0-56) days. Receiving referral from Bulawayo circumcision clinic (RR-1.5 (95% CI-1.3-1.7)) and undergoing circumcision at outreach sites (RR-1.2 (95% CI-1.1-1.3)) were independently associated with not being linked for ASRH services.

For peer review only - http://bmjopen.bmj.com/site/about/guidelines.xhtml

Conclusion: Linkage to ASRH services from VMMC is feasible as one-third referred of VMMC clients were successfully linked. However, there is need to explore reasons for not accessing ASRH services and take corrective actions to improve the linkages.

Keywords: HIV prevention; Adolescent Health; Sexual and Reproductive Health; Youth centres; SORT IT; Operational Research

Article Summary:

Strengths and Limitations:

- First study to have assessed the extent of linkages with Smart-LyncAges project
- No selection bias as we included all the young people (10-24 years) who registered for circumcision services in the VMMC clinics of two project districts
- Routine programmatic data of large cohort was used and thus reflected the field realities in project implementation
- There might have been an underestimation in proportion of young people 'successfully linked' to ASRH services as only those who produced referral slip from VMMC clinic were considered as 'successfully linked'
- Study was conducted only during two months of the year and thus might have failed to account for seasonal variations in seeking ASRH services

For peer review only

INTRODUCTION

Young people (aged 10-24 years) are disproportionately affected by Human Immunodeficiency Virus (HIV) infection with an estimated 3.9 million living with HIV worldwide in 2017.[1,2] Globally, approximately 1600 youths acquire HIV every day and a youth dies every 10 minutes because of AIDS related illness.[1] In contrast to decline in HIV related death rates over years in other age groups, there has been a rise among young people and HIV stands second leading cause of death globally.[3,4] About 84% of young people living with HIV are in Sub-Saharan Africa and it is estimated that an additional 7.4 million young people might become infected with HIV in Sub-Saharan Africa region alone by 2030.[5]

Unprotected sex is the most common route of HIV infection among young people and is largely due to low knowledge on HIV, early sexual debut, multiple sexual partners, and low condom use.[6] This highlights the need for comprehensive sexuality education (CSE) for adolescents before they become sexually active. Realizing this, the United Nations General Assembly Special Session (UNGASS) on HIV in 2001 recommended access to information, youth-specific HIV education and life skills development to at least 95% of young people.[7] Following this, most Sub-Saharan African countries implemented Adolescent Sexual and Reproductive Health (ASRH) services. However, recent studies from this region have reported poor utilization of ASRH services with only about 21%-51% of adolescents accessing it.[8,9] Acknowledging the importance and poor utilisation of ASRH services, the World Health Organization (WHO) recommended to strengthen the linkages between various HIV prevention and ASRH programmes delivered through general health services.[10]

For peer review only

Zimbabwe, a landlocked Sub-Saharan country has a high burden of HIV with an estimated prevalence of 14% among people aged 15 to 49 years.[11] The country has ASRH indicators showing risky behaviours with about 40% girls and 30% boys having sex before age eighteen and 62% of males aged 15-24 not using a condom in their last sexual encounter.[12] HIV prevalence in individuals aged 15–19 years was 3.7% and 4.6% for boys and girls respectively. Among those 20-24 years the HIV prevalence was 8% and 10.8% for boys and girls respectively.[12,13] Thus, since 2009, Zimbabwe has implemented Voluntary Medical Male Circumcision (VMMC) and ASRH services as core strategies to limit HIV burden in young people.

The ASRH programme implemented in 2010, involves the provision of CSE, life skills training, diagnosis and treatment of sexually transmitted infections (STIs) including HIV, family planning counselling, positive masculinity education, vocational training, library services, recreational games and empowerment on rights and responsibility.[14] The ASRH services are delivered at youth friendly clinics or youth centres. There is anecdotal evidence that the majority of young people do not access ASRH services. Whereas, the VMMC programme, implemented as part of combination HIV prevention strategy has a high utilization rate.[15] By 2017, about 80% young boys were covered under VMMC and have had unprecedented contact with the health system. However, the potential of VMMC services as a gateway to additional relevant ASRH services for young people has not been maximized.

The ASRH and VMMC programs have been implemented as two different vertical national health programs, not complementing each other in improving the service utilization. In 2016, the WHO supported the Ministry of Health and Child Care to pilot Smart-LyncAges project to identify approaches to sustain VMMC and improve the

complementarity of both services. Through the project, cross-referral mechanisms were established wherein adolescent boys from VMMC are linked to ASRH programmes and vice versa. Though the Smart-LyncAges project has been piloted as a participatory learning approach since 2016, there was limited quantitative information on the extent of successful linkage of clients between VMMC clinics and ASRH services. This information on extent of successful referral and associated factors was necessary to address any deficiencies in the referral system prior to scale up of the project countrywide. In this regard, the study aimed to assess the proportion of young people referred from VMMC clinics successfully linked for ASRH services and factors associated with 'not linked' in the Smart-LyncAges pilot project sites in Zimbabwe.

For peer review only

METHODOLOGY

Study Design:

This was a cohort study using routinely collected secondary data by the VMMC and ASRH programmes.

Study Setting:

General Setting:

Zimbabwe is a land locked country in Sub Saharan Africa. The country is divided into 10 administrative provinces and has a total of 62 districts. According to the Zimbabwe National Statistics Agency, the country had a projected population of 16,4 million in 2018 and 26% of the total population is adolescents and young adults.[16]

Specific Setting:

The study was conducted in the Bulawayo metropolitan province and Mt Darwin district of Mashonaland Central province, where the VMMC-ASRH linkages project was piloted. Bulawayo is Zimbabwe's second largest city and has a population of approximately 653,000.[16] Mt Darwin is one of seven districts in the Mashonaland Central province with the population of about 213,000. In the district, majority of the population reside in the rural areas.

VMMC services and referral to ASRH

The VMMC programme functions under the National AIDS and TB unit with funding support from external sources mainly The U.S. Presidents Emergency Plan for AIDS Relief (PEPFAR) and Bill and Melinda Gates Foundation (BMGF). The VMMC sites were supported by various non-governmental organizations (NGOs) as implementing partners facilitating

For peer review only

service delivery and community mobilization. The Bulawayo Metropolitan province has two VMMC clinics and the Mount Darwin district has one clinic. The circumcision services were provided at static VMMC clinics and also through outreach clinics in the public health facilities.

Demand generation for VMMC services was created through print and mass media advertisements, school health programmes, road shows and music galas. At community level, the adolescents and young adults were counselled and referred to VMMC clinics by incentivized community mobilizers, peer educators or health workers. The adolescents and young adults registered for ASRH services at youth centres were also referred for VMMC. The adolescents were referred to either static VMMC clinics or outreach clinics based on their convenience.

Upon presentation at a VMMC clinic, the clients were registered with unique VMMC ID number. The clerk documented details of clients in the 'client intake form'. During registration, the clerk issued the referral slip to all the clients who were not already registered for ASRH services. The clients were advised to register themselves for ASRH services in their preferred youth centre and referral slip was provided. The referral slip was created in triplicate; one slip given to the adolescent, one maintained in the referral file at the VMMC clinic and the other slip sent to peer-educator of the service area from where the adolescent has come.

The adolescents registered in the VMMC were offered group education and individual counselling by nurse counsellor. During counselling, importance of registering for ASRH services was emphasized. The nurse counsellors provided HIV testing and pre-operation examination to assess the eligibility for circumcision surgery. Those adolescents with diabetes, keloids and haemophilia were considered as not eligible for surgery. Among

For peer review only

those eligible for surgery, either dorsal slit or forceps guided procedure was performed by either a trained and qualified nurse circumciser or doctor surgeon. Adolescents were advised to make follow-up visits on day-2 and day-7 after the procedure. The adolescents were also advised to visit the clinic in case of any complications. All the details on surgery, follow-up and adverse events were recorded in the 'client intake form'.

ASRH services and registration

The ASRH services were provided at the youth centres. The Bulawayo metropolitan province has seventeen youth centres and the Mount Darwin district has two youth centres. Each youth centre was staffed with a youth health advisor (a registered general nurse), youth facilitator/recreational officer (a social worker) and five peer educators.

Upon arrival at the youth centres, adolescents referred from VMMC submit their VMMC-ASRH referral slips. The youth facilitator/recreational officer registers the adolescents for ASRH services and documents the VMMC ID number in VMMC-ASRH linkages register. The services received by the adolescent during their initial visit were also documented in the VMMC-ASRH linkage register. The referral slip received from the adolescent are stored in the referral box maintained at the youth centre.

Study Population:

All adolescents and young adults (10-24 years) registered in VMMC clinics of Mt Darwin district and Bulawayo city during October and November, 2018 were included in the study. Those who were already registered for ASRH services prior to accessing VMMC services were excluded. Sample size was not calculated and there was no sampling as all the adolescents and young adults in both the pilot districts of Smart-LyncAges project during study reference period were included.

For peer review only

Data Variables, sources of data and data collection:

We extracted details including VMMC identification number, age, education status, mode of referral to VMMC clinic, type of VMMC clinic, HIV status, date of referral to ASRH services, date of registration at VMMC, reason for circumcision, eligibility for circumcision, circumcision status, method of circumcision, status of day-2 post-op visit, status of day-7 post-op visit and adverse events following circumcision within 42 day from the VMMC 'client intake form' maintained at VMMC clinic. The details on registration and date of registration for ASRH services were extracted from VMMC-ARSH linkage register maintained at youth centres. The information on registration for ASRH services extracted from VMMC-ASRH register was validated using the referral slips maintained in the referral box of youth centres. Those individuals linked for ASRH services within three months of receiving referral slip were considered as 'successfully linked' to ASRH services.

The data was extracted in March, 2019 using two separate structured data extraction proformas designed to extract data from VMMC 'client intake form' and VMMC-ARSH linkage register**.** Thus, for each of the study participant registered during October and November, 2018 the linkage status was ascertained only after minimum of 90 days of follow-up. Data extraction proformas were field tested by the principal investigator and modified before data extraction.

Data entry and analysis:

Data was double entered and validated using EpiData entry software (EpiData Association, Odense, Denmark). Two separate data entry structures were used to enter data from two proformas. The two data structures were then merged using unique 'VMMC ID number'. The final merged data file was used for analysis.

For peer review only

Data was analyzed using Stata version 12.0 (STATA Corp., College, TX, USA). Socio-demographic, HIV status and clinical characteristics was summarized using percentages. The age of the participants was summarized with mean and standard deviation (SD). The proportion of participants 'successfully linked' to ASRH was summarized as percentages with 95% confidence interval. The duration between referral to 'successfully linked' to ASRH was summarized with median and inter-quartile range (IQR).

The association between socio-demographic and baseline clinical characteristics with 'not linked for ASRH service' was assessed using bivariate log binomial regression. To assess the independent association, a generalized linear model (Poisson regression) with robust variance estimates was used as log binomial model did not converge.[17,18] Initially, all the variables with p value<0.25 in the bivariate model was included in the multivariate model. Later, the variables with variance inflation factor of more than 10 were removed from the final model and adjusted relative risks (RR) with 95% CI were expressed as measure of association.

Ethics Approval:

The ethics approval was obtained from the Medical Research Council of Zimbabwe (MRCZ/E229) and Ethics Advisory Group of the International Union Against Tuberculosis and Lung Disease, Paris, France (58/18). Permission was sought from the Ministry of Health and Child Care program officials for extracting the routine data collected at VMMC and ASRH centres.

A data sharing statement:

For peer review only

266 Technical appendix, statistical code, and dataset available from the

267 https://www.dropbox.com/sh/cm03olkw3qj8j4x/AAArNAzjweJ1iw0UHq86JIFwa?dl=0

268

269 **Patient and Public Involvement:**

270 Principal Investigator and the data collectors did not interact directly with the young

271 people availing services from VMMC clinics during this retrospective record review. The PI

272 worked with the healthcare staff and peer educators of both the VMMC and ASRH clinics

273 included in the study. Findings from this study will help the Smart-LyncAges project to

274 assess the performance in linkages and also gives insight on deficiencies to be fixed prior to

275 country wide scale-up.

For peer review only

RESULTS

In total 1773 young people (10-24 years) were registered for VMMC services during the study reference period. Of the total, 1478 (83%) had not been previously registered in ASRH clinic and were included in the study. The mean (SD) age of study participants was 13.7 (4.3) years and 427 (28.9%) were out of school. Of the 1478 study participants, 1032 (69.8%) were referred for VMMC by community mobilizer and 1230 (83.2%) reported HIV prevention as the reason for seeking circumcision service. Of the total, 6 (0.3%) had HIV infection. The characteristics of study participants are depicted in **Table-1**.

Of the 1478 study participants, 1461 (99%) were eligible for circumcision surgery and among of those eligible, 1443 (99%) underwent circumcision (**Figure-1**). Of those who underwent circumcision, 477 (33.1%) had the surgery performed at an outreach site. Of the 1443 participants, 1379 (95.6%) and 1025 (71%) attended day 2 and day 7 review visits respectively. In total, 20 (1.4%) developed mild adverse event following circumcision and 3 (0.2%) each developed moderate and severe adverse events (**Table-2**).

Of the 1478 study participants referred for ASRH services, 463 (31.3%, 95% CI-29.0%-33.8%) successfully linked for ASRH services at 'youth centre' within 3 months of referral (**Figure-1**). Among those who were successfully linked, the median (IQR) duration from referral to getting linked at 'youth centre' was 6 (0-56) days. Referral from Bulawayo circumcision clinic (RR-1.5 (95% CI-1.3-1.7)) and undergoing circumcision at outreach sites (RR-1.2 (95% CI-1.1-1.3)) were independently associated with not linked for ASRH services after referral from circumcision clinic. The association between participant's characteristics and failure of referral is shown in **Table-3**.

For peer review only

DISCUSSION

This is the first study assessing the extent of successful referral among adolescents and young men between VMMC clinics and ASRH services under Smart-LyncAges project implemented in Zimbabwe. About 31% of young males (10-24 years) referred from VMMC clinics successfully linked to ASRH services at youth centres within three months of referral. Young people referred from the outreach sites and Bulawayo male circumcision clinic had significantly higher rates of not being linked for ASRH services.

Globally, there were limited studies assessing the linkages between various HIV preventive strategies like VMMC services and ASRH services. A study from Zambia reported that, with enhanced counselling and referral from community, uptake of ASRH services like HIV testing and counselling, family planning counselling and VMMC services was less than 20% within six months.[19] Also, previous reviews have reported that demand generation activities in the community, improving accessibility and quality of ASRH services improve ASRH related knowledge but may not increase the utilization of specific ASRH services.[20–22] The potential reason for underutilization of ASRH services may be that, a preventive strategy may not be considered as an immediate need by young people.[22–24] Considering this, the successful linkage (uptake) of about one in three of the VMMC clients to ASRH services through Smart-LyncAges project (as they produced referral slips) is promising. It would be important to better understand the real and perceived ASRH needs and the relevance of referrals.

The clients referred from Bulawayo MC clinic had slightly higher risk of not being linked to ASRH services. A potential reason for this finding is that, the MC clinic is situated in the eastern suburbs of the city where people of high socio-economic status reside. All the fifteen youth centres were located in western suburbs which are largely populated with

For peer review only

people from low socio economic status. It is possible that, the recreational activities available under ASRH services do not appeal to the technologically savvy urban youths from the elite suburbs. Furthermore, distance to youth centres might have limited the clients from the eastern suburbs to go and register for ASRH services. Similarly, clients accessing VMMC services at outreach clinics were not being linked to ASRH services. The focus of outreach clinics is to conduct surgery in hard to reach areas. Though the referral slips were issued, there would not be space and time to counsel on ASRH services comprehensively. Also, the youth centres in Mt Darwin were mainly located in district centres and might not be accessible to clients of outreach clinics conducted in hard to reach areas. Thus, though not assessed objectively, there might be rural-urban divide in linkages. Although the actual numbers were small, a high proportion of clients ineligible for circumcision or experiencing an adverse event were referred and successfully linked suggesting they have additional service needs.

The study had several strengths. First, the study was conducted within the routine programmatic setting, reflecting the field realities and using routine data. Second, all the young males attending the VMMC clinics during the study reference period were included and thus, there was no sampling bias. Third, the study had large sample size and thus, the estimate of 'successfully linked' was precise. Fourth, the continuous supportive supervision was rendered during the study period to ensure completeness in recording under project setting and thus, the 'missing data' was limited. Fifth, the registration for ASRH services was validated by cross-checking the stored referral slips along with information extracted from VMMC-ASRH linkage register and thus, it enhanced the validity of the outcome ascertainment. Sixth, the STrengthening the Reporting of OBservational Studies in Epidemiology (STROBE) guidelines was used to report the study findings.[25]

For peer review only

The study had a few limitations. First, the registration for ASRH services was ascertained based on the VMMC-ASRH linkage register. Only, those who produced VMMC referral slips during registration were listed in VMMC-ASRH register. Thus, those participants who might have missed their referral slips before registration for ASRH services could have been misclassified as 'not linked'. This could have led to underestimation of percentage of 'successfully' linked. Second, the study was conducted only during two months of the year and thus failed to account for seasonal variations in seeking services. During the festive month of December, the youth centres were closed due to holidays. Also, during December, the youth centres in the Mt Darwin region were closed for two weeks due to cholera outbreak in the region. There might have been an underestimation of 'successfully' linked due to non-functioning of youth centres during study follow-up period. However, as there are no estimates of the extent of underestimation, we failed to account it in estimating the percentage of successfully linked. Third, the pathways for seeking ASRH services and contribution of peer-educators in reaching the ASRH services were not assessed. Thus, the study failed to document the pathways with highest successful linkage rate, which could have helped in replicating the model elsewhere. Fourth, the potential confounders like distance from youth centres, socio-economic status, parents' willingness and adolescents' willingness were not captured and included in the adjusted analysis. Inclusion of these variables could have improved the validity of the model. Fifth, the study represents select group of young people who came for VMMC clinics in the project pilot districts, thus the generalizability of the study results is limited. Lastly, study did not explore the reasons for not being linked for ASRH services among those referred.

The study has a few programmatic implications and recommendations. First, the percentage of 'successfully linked' between VMMC clinics and ASRH services was promising.

For peer review only

The Smart-LyncAges project can be scaled up to improve the utilization of ASRH services. However, there is need for qualitative research to explore the facilitators and barriers for getting linked to ASRH services. This information can help to improve linkages and effectiveness by understanding relevance of linkages to young people`s health needs.

Second, with the existing records maintained under the project, cohort monitoring of the performance of referral system was feasible. The 'proportion of those referred from the facility successfully linked for ASRH services' can be introduced as an indicator in the monthly report of each VMMC clinic. This would enable the programme managers to monitor the performance of referral system better than the absolute number of referred which is being reported now.

Third, the young adolescents seeking VMMC services from outreach clinics at peripheral public health facilities were not getting linked for ASRH services, implying the distance to youth centres was a potential barrier. Solutions should be person centred, such as co-location of ASRH and VMMC services, improved accessibility with services decentralized to peripheral public health facilities and enhanced training of existing general health staff to deliver youth friendly services.

Fourth, about 70% of clients came back to avail follow-up services on day-7 post circumcision. This opportunity can be used to further educate and to reinforce to young people on services available to them as they will be more able to absorb information since the MC procedure has been completed.

CONCLUSION

The rate of young males referred from VMMC clinics and successfully linked for ASRH services was promising. However, there is need to explore the reasons for clients not

For peer review only

accessing the ASRH services, the type of services sought and take corrective actions to improve the same.

ACKNOWLEDGEMENTS

This research was conducted through the Structured Operational Research and Training Initiative (SORT IT), a global partnership led by the Special Programme for Research and Training in Tropical Diseases at the World Health Organization (WHO/TDR). The training model is based on a course developed jointly by the International Union Against Tuberculosis and Lung Disease (The Union) and Medécins sans Frontières (MSF). The specific SORT IT program which resulted in this publication was implemented by the Centre for Operational Research, The Union, Paris, France. Mentorship and the coordination/facilitation of this particular SORT IT workshop was provided through the Centre for Operational Research, The Union, Paris, France; the Department of Tuberculosis and HIV, The Union, Paris, France; The Union, Zimbabwe Office; The Union, South East Asia Office; University of Washington, School of Public Health, Department of Global Health, Seattle, Washington, USA; National Institute for Medical Research, Muhimbili Centre, Dar es Salaam, Tanzania; and AIDS & TB Department, Ministry of Health & Child Care, Harare, Zimbabwe

FUNDING

The training course under which this study was conducted was funded by: the United Kingdom's Department for International Development (DFID); The Global Fund to Fight AIDS, Tuberculosis and Malaria (GFATM) and the World Health Organization (WHO). The WHO also provided funding support for conduct of the study. The funders had no role in

For peer review only

study design, data collection and analysis, decision to publish, or preparation of the manuscript.

AUTHOR CONTRIBUTION

TM was the principal investigator; PT and KT were the SORT IT course mentors; OM, WA, JS, SX and GN were the senior authors. TM, NZ, AM, SM, TT, and RM were involved in data collection; TM, PT and KT analyzed the data and prepared the first draft of the paper. All authors were involved in conception, design, inference of results, providing critical review and approval of the final manuscript.

DISCLOSURE STATEMENT

None of the authors have any competing interests.

For peer review only

REFERENCES

1 Joint United Nations Programme on HIV/AIDS (UNAIDS). Youth and HIV — Mainstreaming a three-lens approach to youth participation. Geneva, Switzerland: 2018.

2 Joint United Nations Programme on HIV/AIDS (UNAIDS). Start Free, Stay Free, AIDS Free. Geneva, Switzerland: 2015.

3 UNICEF. Adolescent deaths from AIDS tripled since 2000 – UNICEF | Press centre | UNICEF. UNICEF. https://www.unicef.org/media/media_86384.html (accessed 8 May 2019).

4 World Health Organization (WHO). WHO | Adolescent deaths and burden of disease. WHO. 2017.https://www.who.int/maternal_child_adolescent/epidemiology/adolescent-deaths-burden-disease/en/ (accessed 8 May 2019).

5 UNICEF. *END AIDS Seventh Stocktaking Report, 2016*. New York, USA: 2016.

6 Joint United Nations Programme on HIV/AIDS (UNAIDS). HIV prevention among adolescent girls and young women. 2016.

7 United Nations General Assembly Special Session on HIV/AIDS. Summary of the Declaration of Commitment on HIV/AIDS - United Nations General Assembly, Special Session on HIV/AIDS, 25-27 June 2001, New York. New York, USA: 2001.

8 Binu W, Marama T, Gerbaba M, *et al.* Sexual and reproductive health services utilization and associated factors among secondary school students in Nekemte town, Ethiopia. doi:10.1186/s12978-018-0501-z

9 Feleke SA, Koye DN, Demssie AF, *et al.* Reproductive health service utilization and associated factors among adolescents (15–19 years old) in Gondar town, Northwest

For peer review only

Ethiopia. *BMC Health Services Research* 2013;**13**:294. doi:10.1186/1472-6963-13-294

10 World Health Organization. The importance of sexual and reproductive health and rights to prevent HIV in adolescent girls and young women in eastern and southern Africa Evidence brief. Geneva, Switzerland: 2017.

11 Ministry of Health and Child Care. ZIMBABWE POPULATION-BASED HIV IMPACT ASSESSMENT ZIMPHIA 2015-16. Harare: 2016.

12 Ministry of Health and Child Care. Zimbabwe Demographic and Health Survey. Harare, Zimbabwe: 2015.

13 Zimbabwe National Statistics Agency. The Zimbabwe Multiple Indicator Cluster. Harae: 2015.

14 Ministry of Health and Child Welfare. National Adolescent Sexual and Reproductive Health Strategy, 2010–2015. Harare, Zimbabwe: 2009.

15 Ministry of Health and Child Care. Accelerated Strategic and Operational Plan 2014-2018. Harare, Zimbabwe: 2019.

16 Zimbabwe National Statistics Agency. ZIMBABWE POPULATION CENSUS 2012. Harare, Zimbabwe: 2012.

17 UCLA Institute for Digital Research and Education. How can I estimate relative risk using glm for common outcomes in cohort studies? | Stata FAQ. Institute for Digital Research and Education. https://stats.idre.ucla.edu/stata/faq/how-can-i-estimate-relative-risk-using-glm-for-common-outcomes-in-cohort-studies/ (accessed 2 Dec 2018).

18 McNutt L-A, Wu C, Xue X, *et al.* Estimating the Relative Risk in Cohort Studies and Clinical Trials of Common Outcomes. *American Journal of Epidemiology* 2003;**157**:940–3. doi:10.1093/aje/kwg074

For peer review only

19 Hewett PC, Nalubamba M, Bozzani F, *et al.* Randomized evaluation and cost-effectiveness of HIV and sexual and reproductive health service referral and linkage models in Zambia. *BMC Public Health* 2016;**16**:785. doi:10.1186/s12889-016-3450-x

20 Mazur A, Brindis CD, Decker MJ. Assessing youth-friendly sexual and reproductive health services: a systematic review. *BMC Health Services Research* 2018;**18**:216. doi:10.1186/s12913-018-2982-4

21 Denno DM, Hoopes AJ, Chandra-Mouli V. Effective Strategies to Provide Adolescent Sexual and Reproductive Health Services and to Increase Demand and Community Support. *Journal of Adolescent Health* 2015;**56**:S22–41. doi:10.1016/J.JADOHEALTH.2014.09.012

22 Chandra-Mouli V, Lane C, Wong S. What Does Not Work in Adolescent Sexual and Reproductive Health: A Review of Evidence on Interventions Commonly Accepted as Best Practices. *Global health, science and practice* 2015;**3**:333–40. doi:10.9745/GHSP-D-15-00126

23 Cassidy C, Bishop A, Steenbeek A, *et al.* Barriers and enablers to sexual health service use among university students: a qualitative descriptive study using the Theoretical Domains Framework and COM-B model. *BMC Health Services Research* 2018;**18**:581. doi:10.1186/s12913-018-3379-0

24 Travers M, O'Uhuru D, Mueller T, *et al.* Implementing Adolescent Sexual and Reproductive Health Clinical Best Practice in the Bronx, New York. *Journal of Adolescent Health* 2019;**64**:376–81. doi:10.1016/j.jadohealth.2018.09.020

25 von Elm E, Altman DG, Egger M, *et al.* The Strengthening the Reporting of Observational Studies in Epidemiology (STROBE) statement: guidelines for reporting observational studies. *The Lancet* 2007;**370**:1453–7. doi:10.1016/S0140-

For peer review only

6736(07)61602-X

For peer review only

508 Table 1: The demographic characteristics, reason for circumcision and HIV status of 509 adolescents and young adults registered for VMMC services during October and 510 November, 2018 in selected health facilities of Zimbabwe, N=1478

Characteristics	Categories	Frequency	(%)
**Total**		**1478**	**(100)**
**Age (in years)**	10-14	980	(66.3)
	15-19	314	(21.2)
	20-24	184	(12.3)
**Education status**	Out of school	427	(28.9)
	Primary education	899	(60.8)
	Secondary education	152	(10.3)
**Health facility**	Mt Darwin	542	(36.7)
	Lobengula MC clinic	587	(39.7)
	Bulawayo MC clinic	349	(23.6)
**Referred by***	Friend or partner	37	(2.5)
	Health worker	216	(14.6)
	Community mobilizer	1032	(69.8)
	Others	139	(9.4)
	Missing	54	(3.7)
**Reasons for circumcision#**	HIV prevention	1230	(83.2)
	Sexual pleasure	248	(16.8)
	STI Prevention	736	(49.8)
	Hygiene	1196	(80.9)
	Socio-cultural reasons	51	(3.5)
**HIV status**	Positive	6	(0.3)
	Negative	1472	(99.7)

513 *Abbreviation:* HIV- Human Immunodeficiency virus; VMMC- Voluntary Medical Male 514 Circumcision; MC- Male Circumcision; STI- Sexually Transmitted Infection
515 * The person who referred the client to VMMC clinic
516 # *Multiple responses are possible*

For peer review only

Table 2: The surgical and follow-up details of adolescents and young adults who underwent circumcision during October and November, 2018 in selected male circumcision clinics of Zimbabwe, N=1443

Characteristics	Categories	Frequency	(%)
**Total**		**1443**	**(100.0)**
**Method of circumcision**	Dorsal slit	1439	(99.7)
	Forceps Guided	4	(0.3)
**Service delivery point***	Static site	966	(66.9)
	Outreach site	477	(33.1)
**Circumciser#**	Nurse practitioner	1418	(98.3)
	Doctor	25	(1.7)
**Attended post-op day-2**	Yes	1379	(95.6)
	No	64	(4.4)
**Attended post-op day-7**	Yes	1025	(71.0)
	No	418	(29.0)
**Adverse event**	None	1417	(98.2)
	Mild	20	(1.4)
	Moderate	3	(0.2)
	Severe	3	(0.2)

* The circumcision in VMMC clinics are considered as static sites. The circumcision done at other public health facilities in a camp mode by staff of VMMC clinics are considered as outreach sites

Circumciser is the one who conducted the circumcision

For peer review only

Table 3: Demographic, HIV status and surgical characteristics associated with not getting linked for ASRH services within 3 months of referral from the selected VMMC health facilities in Zimbabwe, October and November 2018.

Characteristic	Category	Total	Not linked to ASRH, n (%)*	Unadjusted RR (95%. CI)	Adjusted RR (95 % CI)[#]	p value
**Total**		**1478**	**1015 (68.7)**			
**Age (in years)**	10 - 14	980	671 (68.5)	1.1 (1.0-1.2)	1.0 (0.9-1.2)	0.828
	15 - 19	314	200 (63.7)	1	1	
	20 - 24	184	144 (78.3)	1.2 (1.1-1.4)	1.1 (1.0-1.3)	0.168
**Education**	Out of school	427	297 (69.6)	1.1 (1.0-1.3)	1.0 (0.9-1.2)	0.560
	Primary	899	622 (69.2)	1.1 (1.0-1.2)	1.0 (0.9-1.2)	0.632
	Secondary	152	96 (63.2)	1	1	
**Referred By**	Friend/Partner	37	22 (59.5)	1	1	
	Health Worker	216	164 (75.9)	1.3 (1.0-1.7)	1.2 (0.9-1.6)	0.207
	Community mobiliser	1032	694 (67.3)	1.1 (0.9-1.5)	1.1 (0.8-1.5)	0.453
	Others	139	106 (76.3)	1.3 (1.0-1.7)	1.0 (0.8-1.4)	0.849
	Missing	54	29 (53.7)	0.9 (0.6-1.3)	1.2 (0.8-1.6)	0.345
**Health Facility**	Mt Darwin	542	330 (60.9)	1	1	
	Lobengula MC	587	386 (65.8)	1.1 (1.0-1.2)	1.1 (1.0-1.3)	0.087
	Bulawayo MC	349	299 (85.7)	1.4 (1.3-1.5)	1.5 (1.3-1.7)	<0.001
**HIV Status**	Positive	6	5 (83.3)	1.2 (0.8-1.7)	1.3 (0.9-1.9)	0.117
	Negative	1472	1010 (68.6)	1	1	
**Reasons for MC**						
HIV Prevention	Yes	1230	865 (85.2)	1.2 (1.0-1.3)	1.0 (0.9-1.1)	0.800
	No	248	150 (60.5)	1	1	
Sexual Pleasure	Yes	248	158 (63.7)	0.9 (0.8-1.0)	0.9 (0.8-1.1)	0.593
	No	1230	857 (69.7)	1	1	
STI Prevention	Yes	736	523 (71.1)	1.1 (1.0-1.1)	1.0 (0.9-1.1)	0.980
	No	742	492 (66.3)	1	1	
Hygiene	Yes	1196	847(70.8)	1.2 (1.1-1.3)	1.1 (1.0-1.2)	0.183
	No	282	168 (59.6)	1	1	
Socio-cultural	Yes	51	32 (62.8)	0.9 (0.7-1.1)	0.9 (0.7-1.1)	0.407
	No	1427	983 (68.9)	1	1	
**Circumcision[¥]**	Not Eligible	17	3 (17.7)	1	1	
	Circumcised	1443	1003 (69.5)	3.9 (1.4-11)	2.9 (0.6-8.8)	0.062
	Not circumcised	18	9 (50.0)	2.8 (0.9-8.7)	2.3 (0.8-7.1)	0.142
**Circumciser[¥]**	Nurse	1418	989 (69.8)	1.2 (0.9-1.8)	1.2 (0.8-1.8)	0.283
	Doctors	25	14 (56.0)	1	1	
**Service delivery point[¥]**	Static	966	66 (65.8)	1	1	
	Outreach	477	367 (76.9)	1.2 (1.1-1.2)	1.2 (1.1-1.3)	<0.001
**Day-2 follow-up[¥£]**	Yes	1379	965 (70.0)	1.2 (1.0-1.4)		
	No	64	38 (59.4)	1		
**Day-7 follow-up[¥]**	Yes	1025	732 (71.4)	1.1 (1.0-1.2)	0.9 (0.8-1.0)	0.070
	No	418	271 (64.8)	1	1	
**Adverse event[$¥£]**	Yes	26	11 (42.3)	1		
	No	1417	992 (70.0)	1.7 (1.1-2.6)		

For peer review only

552 Abbreviations: ASRH-Adolescent Sexual Reproductive Health; STI-Sexually Transmitted Infection; HIV-Human Immunodeficiency Virus; RR-Relative Risk, CI-Confidence Interval

554 * Row percentage; # Using generalized linear model (Poisson regression); $ Mild, Moderate and Severe were clubbed as 'Yes'; ¥ Applicable only for those who underwent circumcision; £ These factors were removed from the final model as they had variance inflation factor of more than 10

For peer review only

Figure-1: Flow-chart depicting the adolescents eligible for referral to ASRH services, circumcision status and linkage to ASRH services among those registered at selected VMMC clinics of Zimbabwe during October to November 2018

Abbreviation: VMMC- Voluntary Medical Male Circumcision; ASRH- Adolescent Sexual Reproductive Health

For peer review only

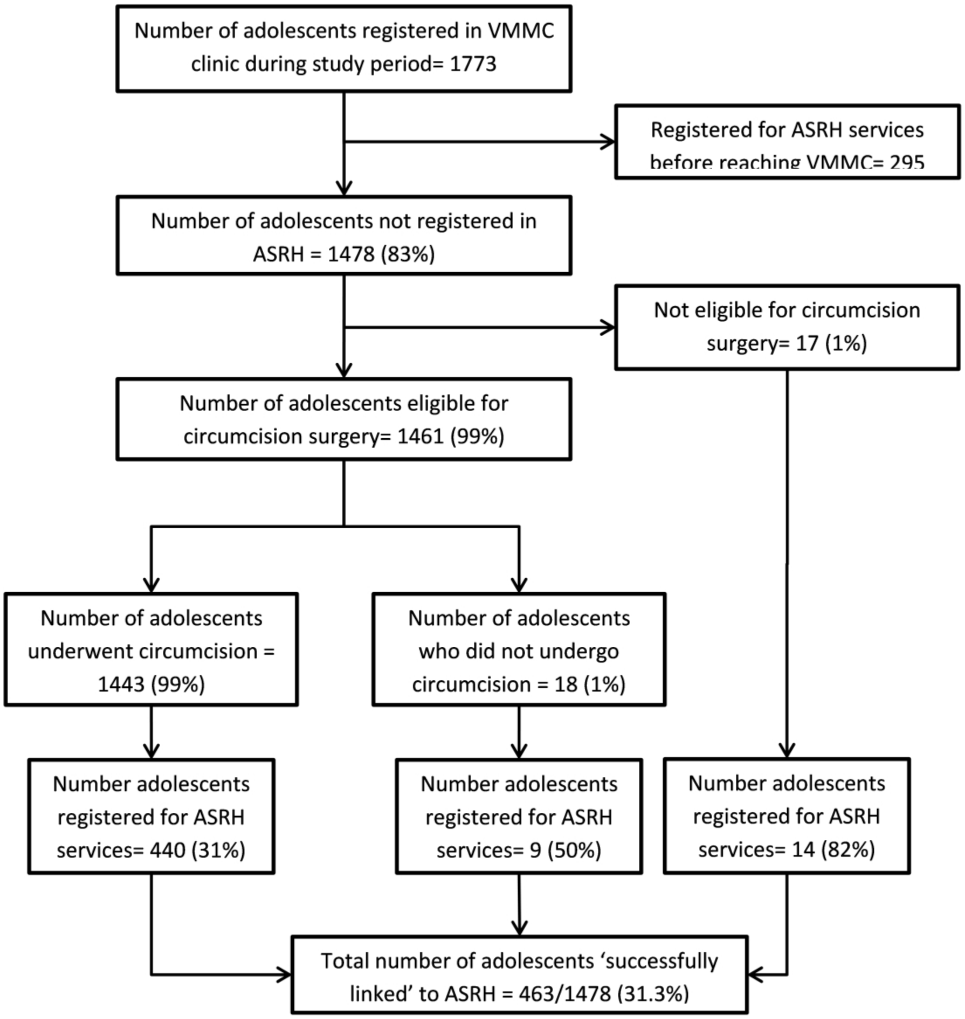

Figure-1: Flow-chart depicting the adolescents eligible for referral to ASRH services, circumcision status and linkage to ASRH services among those registered at selected VMMC clinics of Zimbabwe during October to November 2018

Abbreviation: VMMC- Voluntary Medical Male Circumcision; ASRH- Adolescent Sexual Reproductive Health

1123x1193mm (96 x 96 DPI)

STROBE Statement

Checklist of items that should be included in reports of observational studies

Section/Topic	Item No	Recommendation	Reported on Page No (Line No)
**Title and abstract**	1	(*a*) Indicate the study's design with a commonly used term in the title or the abstract	1(2-3)
		(*b*) Provide in the abstract an informative and balanced summary of what was done and what was found	3(38-67)
**Introduction**			
Background/rationale	2	Explain the scientific background and rationale for the investigation being reported	5-7(85-145)
Objectives	3	State specific objectives, including any prespecified hypotheses	7(142-145)
**Methods**			
Study design	4	Present key elements of study design early in the paper	8(149-150)
Setting	5	Describe the setting, locations, and relevant dates, including periods of recruitment, exposure, follow-up, and data collection	8-10(153-210)
Participants	6	(*a*) *Cohort study*—Give the eligibility criteria, and the sources and methods of selection of participants. Describe methods of follow-up<br>*Case-control study*—Give the eligibility criteria, and the sources and methods of case ascertainment and control selection. Give the rationale for the choice of cases and controls<br>*Cross-sectional study*—Give the eligibility criteria, and the sources and methods of selection of participants	10(213-216)
		(*b*) *Cohort study*—For matched studies, give matching criteria and number of exposed and unexposed<br>*Case-control study*—For matched studies, give matching criteria and the number of controls per case	
Variables	7	Clearly define all outcomes, exposures, predictors, potential confounders, and effect modifiers. Give diagnostic criteria, if applicable	11(220-230)
Data sources/measurement	8*	For each variable of interest, give sources of data and details of methods of assessment (measurement). Describe comparability of assessment methods if there is more than one group	11(231-234)
Bias	9	Describe any efforts to address potential sources of bias	11(237-240, 245-249)
Study size	10	Explain how the study size was arrived at	10(213-216)
Quantitative variables	11	Explain how quantitative variables were handled in the analyses. If applicable, describe which groupings were chosen and why	11,12(241-244)
Statistical methods	12	(*a*) Describe all statistical methods, including those used to control for confounding	11,12(241-249)
		(*b*) Describe any methods used to examine subgroups and interactions	
		(*c*) Explain how missing data were addressed	11(229-230)

For peer review only - http://bmjopen.bmj.com/site/about/guidelines.xhtml

For peer review only

		(*d*) *Cohort study*—If applicable, explain how loss to follow-up was addressed<br>*Case-control study*—If applicable, explain how matching of cases and controls was addressed<br>*Cross-sectional study*—If applicable, describe analytical methods taking account of sampling strategy	NA
		(*e*) Describe any sensitivity analyses	

Section/Topic	Item No	Recommendation	Reported on Page No(Line No)
**Results**			
Participants	13*	(a) Report numbers of individuals at each stage of study—eg numbers potentially eligible, examined for eligibility, confirmed eligible, included in the study, completing follow-up, and analysed	14(277-280)
		(b) Give reasons for non-participation at each stage	
		(c) Consider use of a flow diagram	Figure-1
Descriptive data	14*	(a) Give characteristics of study participants (eg demographic, clinical, social) and information on exposures and potential confounders	14(270-276)
		(b) Indicate number of participants with missing data for each variable of interest	
		(c) *Cohort study*—Summarise follow-up time (eg, average and total amount)	14(283-286)
Outcome data	15*	*Cohort study*—Report numbers of outcome events or summary measures over time	14(283-284)
		Case-control study—Report numbers in each exposure category, or summary measures of exposure	
		Cross-sectional study—Report numbers of outcome events or summary measures	
Main results	16	(*a*) Give unadjusted estimates and, if applicable, confounder-adjusted estimates and their precision (eg, 95% confidence interval). Make clear which confounders were adjusted for and why they were included	14(286-290)
		(*b*) Report category boundaries when continuous variables were categorized	26 (525)
		(*c*) If relevant, consider translating estimates of relative risk into absolute risk for a meaningful time period	
Other analyses	17	Report other analyses done—eg analyses of subgroups and interactions, and sensitivity analyses	
**Discussion**			
Key results	18	Summarise key results with reference to study objectives	15(294-299)
Limitations	19	Discuss limitations of the study, taking into account sources of potential bias or imprecision. Discuss both direction and magnitude of any potential bias	16,17 (338-356)
Interpretation	20	Give a cautious overall interpretation of results considering objectives, limitations, multiplicity of analyses, results from similar studies, and other relevant evidence	15(300-311)
Generalisability	21	Discuss the generalisability (external validity) of the study results	17(355-357)
**Other Information**			

For peer review only - http://bmjopen.bmj.com/site/about/guidelines.xhtml

Funding	22	Give the source of funding and the role of the funders for the present study and, if applicable, for the original study on which the present article is based	19 (407-411)

**Give information separately for cases and controls in case-control studies and, if applicable, for exposed and unexposed groups in cohort and cross-sectional studies.*

Note: An Explanation and Elaboration article discusses each checklist item and gives methodological background and published examples of transparent reporting. The STROBE checklist is best used in conjunction with this article (freely available on the Web sites of PLoS Medicine at http://www.plosmedicine.org/, Annals of Internal Medicine at http://www.annals.org/, and Epidemiology at http://www.epidem.com/). Information on the STROBE Initiative is available at www.strobe-statement.org.

For peer review only

For peer review only - http://bmjopen.bmj.com/site/about/guidelines.xhtml

BMJ Open

Linkage of Voluntary Medical Male Circumcision Clients to Adolescent Sexual and Reproductive Health (ASRH) services through Smart-LyncAges project in Zimbabwe: A cohort study

Journal:	*BMJ Open*
Manuscript ID	bmjopen-2019-033035.R2
Article Type:	Original research
Date Submitted by the Author:	23-Nov-2019
Complete List of Authors:	Makoni, Talent; Ministry of Health and Child Care, AIDS and TB Unit ; World Health Organization Regional Office for Africa<br>Thekkur, Pruthu; International Union Against Tuberculosis and Lung Disease, Centre for Operational Research; The Union South-East Asia Office, Centre for Operational Research<br>Takarinda, Kudakwashe ; Ministry of Health and Child Care<br>Xaba, Sinokuthemba; Ministry of Health and Child Care<br>Ncube , Getrude ; Ministry of Health and Child Care<br>Zwangobani, Nonhlahla; Ministry of Health and Child Care<br>Samuelson, Julia; World Health Organization<br>Mangombe, Aveneni; Ministry of Health and Child Care<br>Mabaya, Simbarashe; World Health Organization Regional Office for Africa<br>Tapera, Talent ; Ministry of Health and Child Care<br>Matambo, Ronald ; The Union Zimbabwe<br>Ameyan, Wole ; World Health Organization<br>Mugurungi, Owen; Ministry of Health and Child Care
**Primary Subject Heading**:	Sexual health
Secondary Subject Heading:	Global health, HIV/AIDS, Sexual health, Reproductive medicine, Epidemiology
Keywords:	HIV & AIDS < INFECTIOUS DISEASES, Adolescent Health, SORT IT, Operational Research, Sexual and Reproductive Health, Youth centres

SCHOLARONE™
Manuscripts

I, the Submitting Author has the right to grant and does grant on behalf of all authors of the Work (as defined in the below author licence), an exclusive licence and/or a non-exclusive licence for contributions from authors who are: i) UK Crown employees; ii) where BMJ has agreed a CC-BY licence shall apply, and/or iii) in accordance with the terms applicable for US Federal Government officers or employees acting as part of their official duties; on a worldwide, perpetual, irrevocable, royalty-free basis to BMJ Publishing Group Ltd ("BMJ") its licensees and where the relevant Journal is co-owned by BMJ to the co-owners of the Journal, to publish the Work in this journal and any other BMJ products and to exploit all rights, as set out in our licence.

The Submitting Author accepts and understands that any supply made under these terms is made by BMJ to the Submitting Author unless you are acting as an employee on behalf of your employer or a postgraduate student of an affiliated institution which is paying any applicable article publishing charge ("APC") for Open Access articles. Where the Submitting Author wishes to make the Work available on an Open Access basis (and intends to pay the relevant APC), the terms of reuse of such Open Access shall be governed by a Creative Commons licence – details of these licences and which Creative Commons licence will apply to this Work are set out in our licence referred to above.

Other than as permitted in any relevant BMJ Author's Self Archiving Policies, I confirm this Work has not been accepted for publication elsewhere, is not being considered for publication elsewhere and does not duplicate material already published. I confirm all authors consent to publication of this Work and authorise the granting of this licence.

For peer review only

Linkage of Voluntary Medical Male Circumcision Clients to Adolescent Sexual and Reproductive Health (ASRH) services through Smart-LyncAges project in Zimbabwe: A cohort study

Manuscript Type: Research Article

Short Running Title: Linkage of VMMC clients to ASRH services in Zimbabwe

Investigators and affiliations

Talent M. Makoni[1], Pruthu Thekkur[2,3], Kudakwashe C. Takarinda[4,5], Sinokuthemba Xaba[5], Getrude Ncube[6], Nonhlahla Zwangobani[7], Julia Sumuelson[8], Aveneni Mangombe[9], Simbarashe Mabaya[10], Talent Tapera[11], Ronald Matambo[12], Wole Ameyan[13], Owen Mugurungi[14]

1. National Voluntary Medical Male Circumcision and Adolescent Sexual Reproductive Health Linkages Officer, Ministry of Health, Harare, Zimbabwe

2. Monitoring and Evaluation Officer, Centre for Operational Research, International Union Against Tuberculosis and Lung Diseases, Paris, France

3. The Union-South East Asia Office, New Delhi, India

4. Senior Operational Research Fellow, Centre for Operational Research, The Union, Zimbabwe

5. National Voluntary Medical Male Circumcision Co-ordinator, Ministry of Health, Harare, Zimbabwe

6. National HIV Prevention Co-ordinator, Ministry of Health, Harare, Zimbabwe

For peer review only

7 Director, Technical Services, Zimbabwe National Family Planning Council, Ministry of Health, Harare, Zimbabwe

8. Nurse Epidemiologist, World Health Organization, Geneva, Switzerland

9. National Adolescent Sexual Reproductive Health Co-ordinator, Ministry of Health, Harare, Zimbabwe

10. National Professional Officer, World Health Organization, Zimbabwe

11. Senior Monitoring and Evaluation Officer, Zvandiri Project, AFRICAID, Zimbabwe

12. Operations Officer, The International Union Against Tuberculosis and Lung Diseases, Zimbabwe

13. Technical Officer, Adolescent HIV, World Health Organization, Geneva

14. Director, HIV and TB Programme, Ministry of Health, Harare, Zimbabwe

Corresponding Author:

Talent M. Makoni,

National Voluntary Medical Male Circumcision and Adolescent Sexual Reproductive Health Linkages Officer,

Ministry of Health, Harare, Zimbabwe

Email ID: talent.makoni32@gmail.com

Manuscript details

Word count of abstract - 300; Word count of manuscript - 3482; Tables - 3; Figures - 1; References – 25

For peer review only

ABSTRACT

Objectives: World Health Organization recommended strengthening the linkages between various HIV prevention programmes and Adolescent Sexual Reproductive Health (ASRH) services. The Smart-LyncAges project piloted in Bulawayo city and Mt Darwin district of Zimbabwe established a referral system to link the Voluntary Medical Male Circumcision (VMMC) clients to ASRH services provided at youth centres. Since its inception in 2016, there has been no assessment of the performance of the referral system. Thus, we aimed to assess the proportion of young (10-24 years) VMMC clients getting 'successfully linked' to ASRH services and factors associated with 'not being linked'.

Design: This was a cohort study using routinely collected secondary data.

Setting: All three VMMC clinics of Mt Darwin district and Bulawayo province.

Primary outcome measures: The proportion of 'successfully linked' was summarized as the percentage with a 95% confidence interval (CI). Adjusted relative risks (aRR) using a generalized linear model was calculated as a measure of association between client characteristics and 'not being linked'.

Results: Of 1773 young people registered for VMMC services, 1478 (83%) were referred for ASRH services as they had not registered for ASRH previously. Of those referred for ASRH services, the mean (SD) age of study participants was 13.7 (4.3) years and 427 (28.9%) were out of school. Of the referred, 463 (31.3%, 95% CI- 30.0-33.8) were 'successfully linked' to ASRH services and the median (IQR) duration for linkage was 6 (0-56) days. Receiving a referral from Bulawayo circumcision clinic (RR-1.5 (95% CI-1.3-1.7)) and undergoing circumcision at outreach sites (RR-1.2 (95% CI-1.1-1.3)) were independently associated with 'not being linked' to ASRH services.

For peer review only

Conclusion: Linkage to ASRH services from VMMC is feasible as one-third VMMC clients referred were successfully linked. However, there is need to explore reasons for not accessing ASRH services and take corrective actions to improve the linkages.

Keywords: HIV prevention; Adolescent Health; Sexual and Reproductive Health; Youth centres; SORT IT; Operational Research

Article Summary:

Strengths and Limitations:

- First study to have assessed the extent of linkages with Smart-LyncAges project
- No selection bias as we included all the young people (10-24 years) who registered for circumcision services in the VMMC clinics of two project districts
- Routine programmatic data of large cohort was used and thus reflected the field realities in project implementation
- There might have been an underestimation in the proportion of young people 'successfully linked' to ASRH services as only those who produced referral slip from VMMC clinic were considered as 'successfully linked'
- Study was conducted only during two months of the year and thus might have failed to account for seasonal variations in seeking ASRH services

For peer review only

INTRODUCTION

Young people (aged 10-24 years) are disproportionately affected by the Human Immunodeficiency Virus (HIV) infection, with an estimated 3.9 million living with HIV worldwide in 2017.[1,2] Globally, approximately 1600 youths acquire HIV every day and a youth dies every 10 minutes because of AIDS-related illness.[1] In contrast to the decline in HIV related death rates over the years in other age groups, there has been a rise among young people.[3,4] About 84% of young people living with HIV are in Sub-Saharan Africa and it is estimated that an additional 7.4 million young people might become infected with HIV in Sub-Saharan Africa region alone by 2030.[5]

Unprotected sex is the most common route of HIV infection among young people and is largely due to low knowledge of HIV, early sexual debut, multiple sexual partners, and low condom use.[6] This highlights the need for comprehensive sexuality education (CSE) for adolescents before they become sexually active. Realizing this, the United Nations General Assembly Special Session (UNGASS) on HIV in 2001 recommended access to information, youth-specific HIV education and life skills development to at least 95% of young people.[7] Following this, most Sub-Saharan African countries implemented Adolescent Sexual and Reproductive Health (ASRH) services. However, recent studies from this region have reported poor utilization of ASRH services, with only about 21%-51% of adolescents accessing it.[8,9] Acknowledging the importance and poor utilisation of ASRH services, the World Health Organization (WHO) recommended strengthening the linkages between various HIV prevention and ASRH programmes delivered through general health services.[10]

Zimbabwe, a landlocked Sub-Saharan country, has a high burden of HIV, with an estimated prevalence of 14% among people aged 15 to 49 years.[11] The country has ASRH

For peer review only

indicators showing risky behaviours with about 40% girls and 30% of boys having sex before age eighteen and 62% of males aged 15-24 not using a condom in their last sexual encounter.[12] HIV prevalence in individuals aged 15–19 years was 3.7% and 4.6% for boys and girls, respectively. Among those 20-24 years, the HIV prevalence was 8% and 10.8% for boys and girls, respectively.[12,13] Thus, since 2009, Zimbabwe has implemented Voluntary Medical Male Circumcision (VMMC) and ASRH services as core strategies to limit the HIV burden in young people.

The ASRH programme implemented in 2010, involves the provision of CSE, life skills training, diagnosis and treatment of sexually transmitted infections (STIs) including HIV, family planning counselling, positive masculinity education, vocational training, library services, recreational games and empowerment on rights and responsibility.[14] The ASRH services are delivered at youth-friendly clinics or youth centres. There is anecdotal evidence that the majority of young people do not access ASRH services. Whereas, the VMMC programme implemented as part of an HIV prevention strategy has a high utilization rate.[15] By 2017, about 80% of young boys were covered under VMMC and have had unprecedented contact with the health system. However, the potential of VMMC services as a gateway to additional relevant ASRH services for young people has not been maximized.

The ASRH and VMMC programs have been implemented as two different vertical national health programs, not complementing each other in improving service utilization. In 2016, the WHO supported the Ministry of Health and Child Care to pilot the Smart-LyncAges project to identify approaches to sustain VMMC and improve the complementarity of both services. Through the project, cross-referral mechanisms were established wherein adolescent boys from VMMC are linked to ASRH programmes and vice versa. Though the Smart-LyncAges project has been piloted as a participatory learning approach since 2016,

For peer review only

there is no quantitative information on the extent of successful linkage of clients between VMMC clinics and ASRH services. The information on the extent of successful linkage and factors associated is failure to linkage is necessary to address any deficiencies in the existing referral system prior to scale-up of the project countrywide. In this regard, the study aimed to assess the proportion of young people referred from VMMC clinics successfully linked for ASRH services and factors associated with 'not being linked' in the Smart-LyncAges pilot project sites in Zimbabwe.

For peer review only

METHODOLOGY

Study Design:

This was a cohort study using routinely collected secondary data by the VMMC and ASRH programmes.

Study Setting:

General Setting:

Zimbabwe is a landlocked country in Sub Saharan Africa. The country is divided into ten administrative provinces and has a total of 62 districts. According to the Zimbabwe National Statistics Agency, the country had a projected population of 16,4 million in 2018 and 26% of the total population is adolescents and young adults.[16]

Specific Setting:

The study was conducted in the Bulawayo metropolitan province and Mt Darwin district of Mashonaland Central province, where the VMMC-ASRH linkages project was piloted. Bulawayo is Zimbabwe's second-largest city and has a population of approximately 653,000.[16] Mt Darwin is one of seven districts in the Mashonaland Central province, with a population of about 213,000. In the district, the majority of the population resides in rural areas.

VMMC services and referral to ASRH

The VMMC programme functions under the National AIDS and TB unit with funding support from external sources, mainly The U.S. Presidents Emergency Plan for AIDS Relief (PEPFAR) and Bill and Melinda Gates Foundation (BMGF). The VMMC sites are supported by various non-governmental organizations (NGOs), which act as implementing partners

For peer review only

facilitating service delivery and community mobilization. The Bulawayo Metropolitan province has two VMMC clinics and the Mount Darwin district has one clinic. The circumcision services are provided at static VMMC clinics and also through outreach clinics in the public health facilities.

Demand generation for VMMC services is done through print and mass media advertisements, school health programmes, roadshows and music galas. At the community level, the community mobilizers, peer educators or health workers counsel and refer adolescents and young adults for VMMC clinics. The adolescents and young adults registered for ASRH services are also referred for VMMC. The referral is made to either static VMMC clinics or outreach clinics based on their convenience.

Upon presentation at a VMMC clinic, the clients are registered with a unique VMMC ID number. The clerk documents the details in the 'client intake form'. During registration, the clerk issues the referral slip to all the clients who are not previously registered for ASRH services. The clients are advised to register themselves for ASRH services in their preferred youth centre and referral slip is provided. The referral slip is created in triplicate, one slip given to the adolescent, one maintained in the referral file at the VMMC clinic and the other slip sent to the peer-educator of the service area from where the adolescent has come.

A nurse counsellor offers group education and individual counselling to clients registered for VMMC. During counselling, the importance of registering for ASRH services is emphasized. The nurse counsellors provide HIV testing and a pre-operation examination to assess the eligibility for circumcision surgery. Those adolescents with diabetes, keloids and haemophilia are considered not eligible for surgery. Among those eligible for surgery, either dorsal slit or forceps guided procedure is performed by either a trained and qualified nurse circumciser or doctor surgeon. Clients are advised to make follow-up visits on day-2 and

For peer review only

day-7 after the procedure. All the details on surgery, follow-up and adverse events are recorded in the 'client intake form'.

ASRH services and registration

The ASRH services are provided at the youth centres. The Bulawayo metropolitan province has seventeen youth centres and the Mount Darwin district has two youth centres. Each youth centre is staffed with a youth health advisor (a registered general nurse), a youth facilitator/recreational officer (a social worker) and five peer educators.

Upon arrival at the youth centres, the VMMC clients submit their VMMC-ASRH referral slips. The youth facilitator/recreational officer registers the adolescents for ASRH services and documents the VMMC ID number in the VMMC-ASRH linkages register. The services received by the adolescent during their initial visit are documented in the VMMC-ASRH linkage register. The referral slip received from the adolescent is stored in the referral box maintained at the youth centre.

Study Population:

All adolescents and young adults (10-24 years) registered in VMMC clinics of Mt Darwin district and Bulawayo city during October and November 2018 were included in the study. Those who were previously registered for ASRH services prior to accessing VMMC services were excluded. The sample size was not calculated and there was no sampling as all the adolescents and young adults in both the pilot districts of the Smart-LyncAges project during study reference period were included.

Data Variables, sources of data and data collection:

We extracted details including VMMC identification number, age, education status, mode of referral to VMMC clinic, type of VMMC clinic, HIV status, date of referral to ASRH

For peer review only

services, date of registration at VMMC, reason for circumcision, eligibility for circumcision, circumcision status, method of circumcision, status of day-2 post-op visit, status of day-7 post-op visit and adverse events following circumcision within 42 day from the VMMC 'client intake form' maintained at VMMC clinic. The details on registration and date of registration for ASRH services were extracted from the VMMC-ARSH linkage register maintained at youth centres. The information on registration for ASRH services extracted from the VMMC-ASRH register was validated using the referral slips maintained in the referral box of youth centres. Those individuals linked for ASRH services within three months of receiving referral slip were considered as 'successfully linked' to ASRH services.

The data was extracted in March 2019 using two separate structured data extraction proformas designed to extract data from VMMC 'client intake form' and VMMC-ARSH linkage register**.** Thus, for each of the study participants registered during October and November 2018, the linkage status was ascertained only after a minimum of 90 days of follow-up. The principal investigator field-tested data extraction proformas and modified them before data extraction.

Data entry and analysis:

Data were double entered and validated using EpiData Entry software (EpiData Association, Odense, Denmark). Two separate data entry structures were used to enter data from two proformas. The two data structures were then merged using the unique 'VMMC ID number'. The final merged data file was used for analysis.

Data were analyzed using Stata version 12.0 (STATA Corp., College, TX, USA). Socio-demographic, HIV status and clinical characteristics were summarized using percentages. The age of the participants was summarized with mean and standard deviation (SD). The

For peer review only

proportion of participant ‘successfully linked’ to ASRH was summarized as percentages with 95% confidence interval. The duration between referral to ‘successfully linked’ to ASRH was summarized with median and inter-quartile range (IQR).

The association between socio-demographic and baseline clinical characteristics with ‘not being linked’ for ASRH service was assessed using bivariate log-binomial regression. A generalized linear model (Poisson regression) with robust variance estimates was used for multivariate regression as the log-binomial model did not converge.[17,18] Initially, all the variables with p value<0.25 in the bivariate model was included in the multivariate model. Later, the variables with a variance inflation factor of more than ten were removed from the final model and adjusted relative risks (aRR) with 95% CI were expressed as measure of association.

Ethics Approval:

The ethics approval was obtained from the Medical Research Council of Zimbabwe (MRCZ/E229) and Ethics Advisory Group of the International Union Against Tuberculosis and Lung Disease, Paris, France (58/18). Permission was sought from the Ministry of Health and Child Care program officials for extracting the routine data collected at VMMC and ASRH centres.

A data sharing statement:

Technical appendix, statistical code, and dataset available from the https://www.dropbox.com/sh/cm03olkw3qj8j4x/AAArNAzjweJ1iw0UHq86JIFwa?dl=0

Patient and Public Involvement:

For peer review only

Principal Investigator and the data collectors did not interact directly with the young people availing services from VMMC clinics during this retrospective record review. The PI worked with the healthcare staff and peer educators of both the VMMC and ASRH clinics included in the study. Findings from this study will help the Smart-LyncAges project to assess the performance in linkages and also gives insight on deficiencies to be fixed prior to country wide scale-up.

For peer review only

RESULTS

In total, 1773 young people (10-24 years) were registered for VMMC services during the study reference period. Of the total, 1478 (83%) had not been previously registered in the ASRH clinic and were included in the study. The mean (SD) age of study participants was 13.7 (4.3) years and 427 (28.9%) were out of school. Of the 1478 study participants, 1032 (69.8%) were referred for VMMC by community mobilizer and 1230 (83.2%) reported HIV prevention as the reason for seeking circumcision service. Of the total, 6 (0.3%) had HIV infection. The characteristics of study participants are depicted in **Table-1**.

Of the 1478 study participants, 1461 (99%) were eligible for circumcision surgery, of which, 1443 (99%) underwent circumcision (**Figure-1**). Of those who underwent circumcision, 477 (33.1%) had the surgery performed at an outreach site. Of the 1443 participants, 1379 (95.6%) and 1025 (71%) attended day-2 and day-7 review visits, respectively. In total, 20 (1.4%) developed mild adverse events following circumcision and 3 (0.2%) each developed moderate and severe adverse events (**Table-2**).

Of the 1478 study participants referred for ASRH services, 463 (31.3%, 95% CI-29.0%-33.8%) successfully linked for ASRH services at 'youth centre' within three months of referral (**Figure-1**). Among those who were successfully linked, the median (IQR) duration from referral to getting linked at 'youth centre' was 6 (0-56) days. Referral from Bulawayo circumcision clinic (RR-1.5 (95% CI-1.3-1.7)) and undergoing circumcision at outreach sites (RR-1.2 (95% CI-1.1-1.3)) were independently associated with not being linked for ASRH services after referral from circumcision clinic. The AIC and BIC of the model were 2799.2 and 2926.3, respectively. The LR test was significant compared to constant model (p value= 0.014). The association between participant's characteristics and not being linked is shown in **Table-3**.

For peer review only

DISCUSSION

This is the first study assessing the extent of successful referral among adolescents and young men between VMMC clinics and ASRH services under the Smart-LyncAges project implemented in Zimbabwe. About 31% of young males (10-24 years) referred from VMMC clinics successfully linked to ASRH services at youth centres within three months of referral. Young people referred from the outreach sites and Bulawayo male circumcision clinic had significantly higher rates of not being linked for ASRH services.

Globally, there is limited literature on linkages between various HIV preventive strategies like VMMC services and ASRH services. A study from Zambia reported that, with enhanced counselling and referral from the community, uptake of ASRH services like HIV testing and counselling, family planning counselling and VMMC services was less than 20% within six months.[19] Also, previous reviews have reported that demand generation activities in the community, improving accessibility and quality of ASRH services, improve ASRH related knowledge but may not increase the utilization of specific ASRH services.[20–22] The potential reason for underutilization is that the preventive strategy like ASRH may not be considered as an immediate need by young people.[22–24] Considering this, the successful linkage (uptake) of about one in three of the VMMC clients to ASRH services through the Smart-LyncAges project (as they produced referral slips) is promising.

The clients referred from the Bulawayo MC clinic had a slightly higher risk of not being linked to ASRH services. A potential reason for this finding is that the MC clinic is situated in the eastern suburbs of the city where people of high socio-economic status reside. All the fifteen youth centres were located in western suburbs, which are largely populated with people from low socio-economic status. It is possible that the recreational activities available under ASRH services do not appeal to the technologically savvy urban

For peer review only

youths from the elite suburbs. Furthermore, distance to youth centres might have limited the clients from the eastern suburbs to go and register for ASRH services. Similarly, clients accessing VMMC services at outreach clinics were not being linked to ASRH services. The focus of outreach clinics is to conduct surgery in hard to reach areas. Though the referral slips were issued, there would not be space and time to counsel on ASRH services comprehensively. Also, the youth centres in Mt Darwin were mainly located in district centres and might not be accessible to clients of outreach clinics conducted in hard to reach areas. Thus, though not assessed objectively, there might be a rural-urban divide in linkages. Although the actual numbers were small, a high proportion of clients ineligible for circumcision or experiencing an adverse event were referred and successfully linked, suggesting they have additional service needs.

The study had several strengths. First, the study was conducted within the routine programmatic setting, reflecting the field realities and using routine data. Second, all the young males attending the VMMC clinics during the study reference period were included and thus, there was no sampling bias. Third, the study had a large sample size and thus, the estimate of 'successfully linked' was precise. Fourth, the continuous supportive supervision was rendered during the study period to ensure completeness in recording under the project setting and thus, the 'missing data' was limited. Fifth, the registration for ASRH services was validated by cross-checking the stored referral slips along with information extracted from the VMMC-ASRH linkage register and thus, it enhanced the validity of the outcome ascertainment. Sixth, the STrengthening the Reporting of OBservational Studies in Epidemiology (STROBE) guidelines was used to report the study findings.[25]

The study had a few limitations. First, the registration for ASRH services was ascertained based on the VMMC-ASRH linkage register. Only those who produced VMMC

referral slips during registration were listed in the VMMC-ASRH register. Thus, those participants who might have missed their referral slips before registration for ASRH services could have been misclassified as 'not linked'. This could have led to the underestimation of the percentage of 'successfully' linked. Second, the study was conducted only during two months of the year and thus failed to account for seasonal variations in seeking services. During the festive month of December, the youth centres were closed due to holidays. Also, during December, the youth centres in the Mt Darwin region were closed for two weeks due to the cholera outbreak in the region. There might have been an underestimation of 'successfully' linked due to the non-functioning of youth centres during the study follow-up period. However, as there are no estimates of the extent of underestimation, we failed to account for it in estimating the percentage of successfully linked. Third, the pathways for seeking ASRH services and the contribution of peer-educators in reaching the ASRH services were not assessed. Thus, the study failed to document the pathways with the highest successful linkage rate, which could have helped in replicating the model elsewhere. Fourth, the potential confounders like distance from youth centres, socio-economic status, parents' willingness and adolescents' willingness were not captured and included in the adjusted analysis. The inclusion of these variables could have improved the validity of the model. Fifth, the study represents select group of young people who came for VMMC clinics in the project pilot districts. Thus, the generalizability of the study results is limited. Lastly, the study did not explore the reasons for not being linked to ASRH services among those referred.

The study has a few programmatic implications and recommendations. First, the percentage of 'successfully linked' between VMMC clinics and ASRH services was promising. The Smart-LyncAges project can be scaled up to improve the utilization of ASRH services.

For peer review only

However, there is a need for qualitative research to explore the facilitators and barriers to getting linked to ASRH services. This information can help to improve the linkages and effectiveness of the programme.

Second, with the existing records maintained under the project, cohort monitoring of the performance of the referral system was feasible. The 'proportion of those referred from the facility successfully linked for ASRH services' can be introduced as an indicator in the monthly report of each VMMC clinic. This would enable the programme managers to monitor the performance of the referral system better than the absolute number of referred and linked, which is currently being monitored.

Third, the young adolescents seeking VMMC services from outreach clinics at peripheral public health facilities were not getting linked for ASRH services, implying the distance to youth centres was a potential barrier. Solutions should be person-centred, such as co-location of ASRH and VMMC services, improved accessibility with services decentralized to peripheral public health facilities and enhanced training of existing general health staff to deliver youth-friendly services.

Fourth, about 70% of clients came back to avail follow-up services on day-7 post circumcision. This opportunity can be used to educate further and to reinforce the importance of ASRH services. The clients might be more responsive to the information since the circumcision procedure is already completed.

CONCLUSION

The rate of young males referred from VMMC clinics and successfully linked to ASRH services was promising. Those referred from the outreach sites and clinics in Bulawayo had significantly higher rates of 'not being linked'. However, there is a need to explore the

For peer review only

reasons for clients not accessing the ASRH services and take corrective actions to improve the linkages.

ACKNOWLEDGEMENTS

This research was conducted through the Structured Operational Research and Training Initiative (SORT IT), a global partnership led by the Special Programme for Research and Training in Tropical Diseases at the World Health Organization (WHO/TDR). The training model is based on a course developed jointly by the International Union Against Tuberculosis and Lung Disease (The Union) and Medécins sans Frontières (MSF). The specific SORT IT program which resulted in this publication was implemented by the Centre for Operational Research, The Union, Paris, France. Mentorship and the coordination/facilitation of this particular SORT IT workshop was provided through the Centre for Operational Research, The Union, Paris, France; the Department of Tuberculosis and HIV, The Union, Paris, France; The Union, Zimbabwe Office; The Union, South East Asia Office; University of Washington, School of Public Health, Department of Global Health, Seattle, Washington, USA; National Institute for Medical Research, Muhimbili Centre, Dar es Salaam, Tanzania; and AIDS & TB Department, Ministry of Health & Child Care, Harare, Zimbabwe

FUNDING

The training course under which this study was conducted was funded by: the United Kingdom's Department for International Development (DFID); The Global Fund to Fight AIDS, Tuberculosis and Malaria (GFATM) and the World Health Organization (WHO). The WHO also provided funding support for conduct of the study. The funders had no role in

For peer review only

study design, data collection and analysis, decision to publish, or preparation of the manuscript.

AUTHOR CONTRIBUTION

TM was the principal investigator; PT and KT were the SORT IT course mentors; OM, WA, JS, SX and GN were the senior authors. TM, NZ, AM, SM, TT, and RM were involved in data collection; TM, PT and KT analyzed the data and prepared the first draft of the paper. All authors were involved in conception, design, inference of results, providing critical review and approval of the final manuscript.

DISCLOSURE STATEMENT

None of the authors have any competing interests.

For peer review only

REFERENCES

1 Joint United Nations Programme on HIV/AIDS (UNAIDS). Youth and HIV — Mainstreaming a three-lens approach to youth participation. Geneva, Switzerland: 2018.

2 Joint United Nations Programme on HIV/AIDS (UNAIDS). Start Free, Stay Free, AIDS Free. Geneva, Switzerland: 2015.

3 UNICEF. Adolescent deaths from AIDS tripled since 2000 – UNICEF | Press centre | UNICEF. UNICEF. https://www.unicef.org/media/media_86384.html (accessed 8 May 2019).

4 World Health Organization (WHO). WHO | Adolescent deaths and burden of disease. WHO. 2017.https://www.who.int/maternal_child_adolescent/epidemiology/adolescent-deaths-burden-disease/en/ (accessed 8 May 2019).

5 UNICEF. *END AIDS Seventh Stocktaking Report, 2016*. New York, USA: 2016.

6 Joint United Nations Programme on HIV/AIDS (UNAIDS). HIV prevention among adolescent girls and young women. 2016.

7 United Nations General Assembly Special Session on HIV/AIDS. Summary of the Declaration of Commitment on HIV/AIDS - United Nations General Assembly, Special Session on HIV/AIDS, 25-27 June 2001, New York. New York, USA: 2001.

8 Binu W, Marama T, Gerbaba M, *et al.* Sexual and reproductive health services utilization and associated factors among secondary school students in Nekemte town, Ethiopia. doi:10.1186/s12978-018-0501-z

9 Feleke SA, Koye DN, Demssie AF, *et al.* Reproductive health service utilization and associated factors among adolescents (15–19 years old) in Gondar town, Northwest

For peer review only

Ethiopia. *BMC Health Services Research* 2013;**13**:294. doi:10.1186/1472-6963-13-294

10 World Health Organization. The importance of sexual and reproductive health and rights to prevent HIV in adolescent girls and young women in eastern and southern Africa Evidence brief. Geneva, Switzerland: 2017.

11 Ministry of Health and Child Care. ZIMBABWE POPULATION-BASED HIV IMPACT ASSESSMENT ZIMPHIA 2015-16. Harare: 2016.

12 Ministry of Health and Child Care. Zimbabwe Demographic and Health Survey. Harare, Zimbabwe: 2015.

13 Zimbabwe National Statistics Agency. The Zimbabwe Multiple Indicator Cluster. Harae: 2015.

14 Ministry of Health and Child Welfare. National Adolescent Sexual and Reproductive Health Strategy, 2010–2015. Harare, Zimbabwe: 2009.

15 Ministry of Health and Child Care. Accelerated Strategic and Operational Plan 2014-2018. Harare, Zimbabwe: 2019.

16 Zimbabwe National Statistics Agency. ZIMBABWE POPULATION CENSUS 2012. Harare, Zimbabwe: 2012.

17 UCLA Institute for Digital Research and Education. How can I estimate relative risk using glm for common outcomes in cohort studies? | Stata FAQ. Institute for Digital Research and Education. https://stats.idre.ucla.edu/stata/faq/how-can-i-estimate-relative-risk-using-glm-for-common-outcomes-in-cohort-studies/ (accessed 2 Dec 2018).

18 McNutt L-A, Wu C, Xue X, *et al.* Estimating the Relative Risk in Cohort Studies and Clinical Trials of Common Outcomes. *American Journal of Epidemiology* 2003;**157**:940–3. doi:10.1093/aje/kwg074

For peer review only

19 Hewett PC, Nalubamba M, Bozzani F, *et al.* Randomized evaluation and cost-effectiveness of HIV and sexual and reproductive health service referral and linkage models in Zambia. *BMC Public Health* 2016;**16**:785. doi:10.1186/s12889-016-3450-x

20 Mazur A, Brindis CD, Decker MJ. Assessing youth-friendly sexual and reproductive health services: a systematic review. *BMC Health Services Research* 2018;**18**:216. doi:10.1186/s12913-018-2982-4

21 Denno DM, Hoopes AJ, Chandra-Mouli V. Effective Strategies to Provide Adolescent Sexual and Reproductive Health Services and to Increase Demand and Community Support. *Journal of Adolescent Health* 2015;**56**:S22–41. doi:10.1016/J.JADOHEALTH.2014.09.012

22 Chandra-Mouli V, Lane C, Wong S. What Does Not Work in Adolescent Sexual and Reproductive Health: A Review of Evidence on Interventions Commonly Accepted as Best Practices. *Global health, science and practice* 2015;**3**:333–40. doi:10.9745/GHSP-D-15-00126

23 Cassidy C, Bishop A, Steenbeek A, *et al.* Barriers and enablers to sexual health service use among university students: a qualitative descriptive study using the Theoretical Domains Framework and COM-B model. *BMC Health Services Research* 2018;**18**:581. doi:10.1186/s12913-018-3379-0

24 Travers M, O'Uhuru D, Mueller T, *et al.* Implementing Adolescent Sexual and Reproductive Health Clinical Best Practice in the Bronx, New York. *Journal of Adolescent Health* 2019;**64**:376–81. doi:10.1016/j.jadohealth.2018.09.020

25 von Elm E, Altman DG, Egger M, *et al.* The Strengthening the Reporting of Observational Studies in Epidemiology (STROBE) statement: guidelines for reporting observational studies. *The Lancet* 2007;**370**:1453–7. doi:10.1016/S0140-

For peer review only

6736(07)61602-X

For peer review only

Table 1: The demographic characteristics, reason for circumcision and HIV status of adolescents and young adults registered for VMMC services during October and November, 2018 in selected health facilities of Zimbabwe, N=1478

Characteristics	Categories	Frequency	(%)
**Total**		**1478**	**(100)**
**Age (in years)**	10-14	980	(66.3)
	15-19	314	(21.2)
	20-24	184	(12.3)
**Education status**	Out of school	427	(28.9)
	Primary education	899	(60.8)
	Secondary education	152	(10.3)
**Health facility**	Mt Darwin	542	(36.7)
	Lobengula MC clinic	587	(39.7)
	Bulawayo MC clinic	349	(23.6)
**Referred by***	Friend or partner	37	(2.5)
	Health worker	216	(14.6)
	Community mobilizer	1032	(69.8)
	Others	139	(9.4)
	Missing	54	(3.7)
**Reasons for circumcision#**	HIV prevention	1230	(83.2)
	Sexual pleasure	248	(16.8)
	STI Prevention	736	(49.8)
	Hygiene	1196	(80.9)
	Socio-cultural reasons	51	(3.5)
**HIV status**	Positive	6	(0.3)
	Negative	1472	(99.7)

Abbreviation: HIV- Human Immunodeficiency virus; VMMC- Voluntary Medical Male Circumcision; MC- Male Circumcision; STI- Sexually Transmitted Infection

* The person who referred the client to VMMC clinic

Multiple responses are possible

For peer review only

Table 2: The surgical and follow-up details of adolescents and young adults who underwent circumcision during October and November, 2018 in selected male circumcision clinics of Zimbabwe, N=1443

Characteristics	Categories	Frequency	(%)
**Total**		**1443**	**(100.0)**
**Method of circumcision**	Dorsal slit	1439	(99.7)
	Forceps Guided	4	(0.3)
**Service delivery point***	Static site	966	(66.9)
	Outreach site	477	(33.1)
**Circumciser#**	Nurse practitioner	1418	(98.3)
	Doctor	25	(1.7)
**Attended post-op day-2**	Yes	1379	(95.6)
	No	64	(4.4)
**Attended post-op day-7**	Yes	1025	(71.0)
	No	418	(29.0)
**Adverse event**	None	1417	(98.2)
	Mild	20	(1.4)
	Moderate	3	(0.2)
	Severe	3	(0.2)

* The circumcision in VMMC clinics are considered as static sites. The circumcision done at other public health facilities in a camp mode by staff of VMMC clinics are considered as outreach sites

Circumciser is the one who conducted the circumcision

For peer review only

Table 3: Demographic, HIV status and surgical characteristics associated with not getting linked for ASRH services within 3 months of referral from the selected VMMC health facilities in Zimbabwe, October and November 2018.

Characteristic	Category	Total	Not linked to ASRH, n (%)*	Linked to ASRH, n (%)	Unadjusted RR (95%. CI)	Adjusted RR (95 % CI)#	p value
**Total**		**1478**	**1015 (68.7)**	**463 (31.3)**			
**Age (in years)**	10 - 14	980	671 (68.5)	309 (31.5)	1.1 (1.0-1.2)	1.0 (0.9-1.2)	0.828
	15 - 19	314	200 (63.7)	114 (36.3)	1	1	
	20 - 24	184	144 (78.3)	40 (21.7)	1.2 (1.1-1.4)	1.1 (1.0-1.3)	0.168
**Education**	Out of school	427	297 (69.6)	130 (30.4)	1.1 (1.0-1.3)	1.0 (0.9-1.2)	0.560
	Primary	899	622 (69.2)	277 (30.8)	1.1 (1.0-1.2)	1.0 (0.9-1.2)	0.632
	Secondary	152	96 (63.2)	56 (36.8)	1	1	
**Referred By**	Friend/Partner	37	22 (59.5)	15 (40.5)	1	1	
	Health Worker	216	164 (75.9)	52 (24.1)	1.3 (1.0-1.7)	1.2 (0.9-1.6)	0.207
	Community mobiliser	1032	694 (67.3)	338 (32.7)	1.1 (0.9-1.5)	1.1 (0.8-1.5)	0.453
	Others	139	106 (76.3)	33 (23.7)	1.3 (1.0-1.7)	1.0 (0.8-1.4)	0.849
	Missing	54	29 (53.7)	25 (46.3)	0.9 (0.6-1.3)	1.2 (0.8-1.6)	0.345
**Health Facility**	Mt Darwin	542	330 (60.9)	212 (39.1)	1	1	
	Lobengula MC	587	386 (65.8)	201 (34.2)	1.1 (1.0-1.2)	1.1 (1.0-1.3)	0.087
	Bulawayo MC	349	299 (85.7)	50 (14.3)	1.4 (1.3-1.5)	1.5 (1.3-1.7)	<0.001
**HIV Status**	Positive	6	5 (83.3)	1 (16.7)	1.2 (0.8-1.7)	1.3 (0.9-1.9)	0.117
	Negative	1472	1010 (68.6)	462 (31.4)	1	1	
**Reasons for MC**							
HIV Prevention	Yes	1230	865 (85.2)	365 (14.8)	1.2 (1.0-1.3)	1.0 (0.9-1.1)	0.800
	No	248	150 (60.5)	98 (39.5)	1	1	
Sexual Pleasure	Yes	248	158 (63.7)	90 (36.3)	0.9 (0.8-1.0)	0.9 (0.8-1.1)	0.593
	No	1230	857 (69.7)	373 (30.3)	1	1	
STI Prevention	Yes	736	523 (71.1)	213 (28.9)	1.1 (1.0-1.1)	1.0 (0.9-1.1)	0.980
	No	742	492 (66.3)	250 (33.7)	1	1	
Hygiene	Yes	1196	847 (70.8)	349 (29.2)	1.2 (1.1-1.3)	1.1 (1.0-1.2)	0.183
	No	282	168 (59.6)	114 (40.4)	1	1	

For peer review only

Socio-cultural	Yes	51	32 (62.8)	19 (37.2)	0.9 (0.7-1.1)	0.9 (0.7-1.1)	0.407
	No	1427	983 (68.9)	444 (31.1)	1	1	
**Circumcision[¥]**	Not Eligible	17	3 (17.7)	14 (82.3)	1	1	
	Circumcised	1443	1003 (69.5)	440 (30.5)	3.9 (1.4-11)	2.9 (0.6-8.8)	0.062
	Not circumcised	18	9 (50.0)	9 (50.0)	2.8 (0.9-8.7)	2.3 (0.8-7.1)	0.142
**Circumciser[¥]**	Nurse	1418	989 (69.8)	429 (30.2)	1.2 (0.9-1.8)	1.2 (0.8-1.8)	0.283
	Doctors	25	14 (56.0)	11 (44.0)	1	1	
**Service delivery point[¥]**	Static	966	66 (65.8)	900 (34.2)	1	1	
	Outreach	477	367 (76.9)	110 (23.1)	1.2 (1.1-1.2)	1.2 (1.1-1.3)	<0.001
**Day-2 follow-up[¥£]**	Yes	1379	965 (70.0)	414 (30.0)	1.2 (1.0-1.4)		
	No	64	38 (59.4)	26 (40.6)	1		
**Day-7 follow-up[¥]**	Yes	1025	732 (71.4)	293 (28.6)	1.1 (1.0-1.2)	0.9 (0.8-1.0)	0.070
	No	418	271 (64.8)	147 (35.2)	1	1	
**Adverse event[$¥£]**	Yes	26	11 (42.3)	15 (57.7)	1		
	No	1417	992 (70.0)	425 (30.0)	1.7 (1.1-2.6)		

Abbreviations: ASRH-Adolescent Sexual Reproductive Health; STI-Sexually Transmitted Infection; HIV-Human Immunodeficiency Virus; RR-Relative Risk, CI-Confidence Interval

* Row percentage; [#] Using generalized linear model (Poisson regression); [$] Mild, Moderate and Severe were clubbed as ‘Yes’; [¥] Applicable only for those who underwent circumcision; [£] These factors were removed from the final model as they had variance inflation factor of more than 10

For peer review only

Figure-1: Flow-chart depicting the adolescents eligible for referral to ASRH services, circumcision status and linkage to ASRH services among those registered at selected VMMC clinics of Zimbabwe during October to November 2018

Abbreviation: VMMC- Voluntary Medical Male Circumcision; ASRH- Adolescent Sexual Reproductive Health

For peer review only

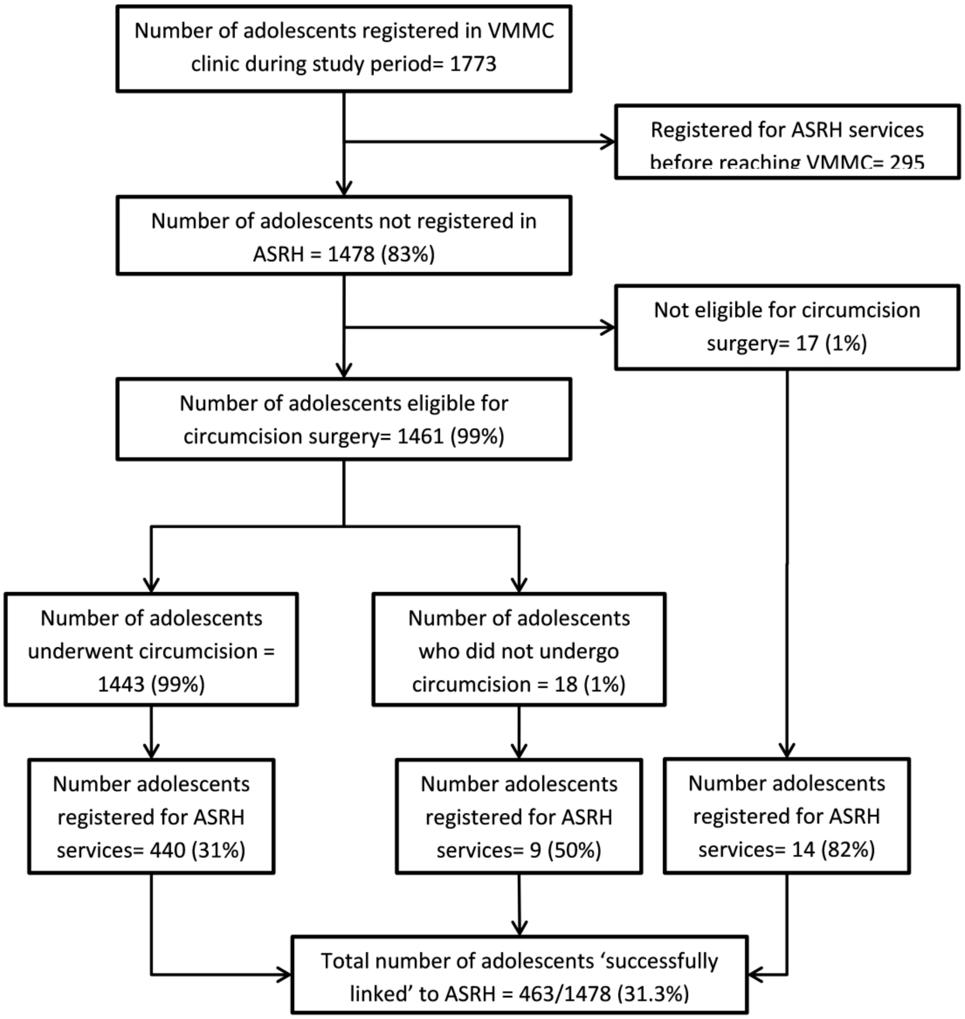

Figure-1: Flow-chart depicting the adolescents eligible for referral to ASRH services, circumcision status and linkage to ASRH services among those registered at selected VMMC clinics of Zimbabwe during October to November 2018

Abbreviation: VMMC- Voluntary Medical Male Circumcision; ASRH- Adolescent Sexual Reproductive Health

1123x1193mm (96 x 96 DPI)

STROBE Statement

Checklist of items that should be included in reports of observational studies

Section/Topic	Item No	Recommendation	Reported on Page No (Line No)
**Title and abstract**	1	(*a*) Indicate the study's design with a commonly used term in the title or the abstract	1(2-3)
		(*b*) Provide in the abstract an informative and balanced summary of what was done and what was found	3(38-67)
**Introduction**			
Background/rationale	2	Explain the scientific background and rationale for the investigation being reported	5-7(85-145)
Objectives	3	State specific objectives, including any prespecified hypotheses	7(142-145)
**Methods**			
Study design	4	Present key elements of study design early in the paper	8(149-150)
Setting	5	Describe the setting, locations, and relevant dates, including periods of recruitment, exposure, follow-up, and data collection	8-10(153-210)
Participants	6	(*a*) *Cohort study*—Give the eligibility criteria, and the sources and methods of selection of participants. Describe methods of follow-up<br>*Case-control study*—Give the eligibility criteria, and the sources and methods of case ascertainment and control selection. Give the rationale for the choice of cases and controls<br>*Cross-sectional study*—Give the eligibility criteria, and the sources and methods of selection of participants	10(213-216)
		(*b*) *Cohort study*—For matched studies, give matching criteria and number of exposed and unexposed<br>*Case-control study*—For matched studies, give matching criteria and the number of controls per case	
Variables	7	Clearly define all outcomes, exposures, predictors, potential confounders, and effect modifiers. Give diagnostic criteria, if applicable	11(220-230)
Data sources/measurement	8*	For each variable of interest, give sources of data and details of methods of assessment (measurement). Describe comparability of assessment methods if there is more than one group	11(231-234)
Bias	9	Describe any efforts to address potential sources of bias	11(237-240, 245-249)
Study size	10	Explain how the study size was arrived at	10(213-216)
Quantitative variables	11	Explain how quantitative variables were handled in the analyses. If applicable, describe which groupings were chosen and why	11,12(241-244)
Statistical methods	12	(*a*) Describe all statistical methods, including those used to control for confounding	11,12(241-249)
		(*b*) Describe any methods used to examine subgroups and interactions	
		(*c*) Explain how missing data were addressed	11(229-230)

For peer review only - http://bmjopen.bmj.com/site/about/guidelines.xhtml

		(*d*) *Cohort study*—If applicable, explain how loss to follow-up was addressed<br>*Case-control study*—If applicable, explain how matching of cases and controls was addressed<br>*Cross-sectional study*—If applicable, describe analytical methods taking account of sampling strategy	NA
		(*e*) Describe any sensitivity analyses	

Section/Topic	Item No	Recommendation	Reported on Page No(Line No)
**Results**			
Participants	13*	(a) Report numbers of individuals at each stage of study—eg numbers potentially eligible, examined for eligibility, confirmed eligible, included in the study, completing follow-up, and analysed	14(277-280)
		(b) Give reasons for non-participation at each stage	
		(c) Consider use of a flow diagram	Figure-1
Descriptive data	14*	(a) Give characteristics of study participants (eg demographic, clinical, social) and information on exposures and potential confounders	14(270-276)
		(b) Indicate number of participants with missing data for each variable of interest	
		(c) *Cohort study*—Summarise follow-up time (eg, average and total amount)	14(283-286)
Outcome data	15*	*Cohort study*—Report numbers of outcome events or summary measures over time	14(283-284)
		Case-control study—Report numbers in each exposure category, or summary measures of exposure	
		Cross-sectional study—Report numbers of outcome events or summary measures	
Main results	16	(*a*) Give unadjusted estimates and, if applicable, confounder-adjusted estimates and their precision (eg, 95% confidence interval). Make clear which confounders were adjusted for and why they were included	14(286-290)
		(*b*) Report category boundaries when continuous variables were categorized	26 (525)
		(*c*) If relevant, consider translating estimates of relative risk into absolute risk for a meaningful time period	
Other analyses	17	Report other analyses done—eg analyses of subgroups and interactions, and sensitivity analyses	
**Discussion**			
Key results	18	Summarise key results with reference to study objectives	15(294-299)
Limitations	19	Discuss limitations of the study, taking into account sources of potential bias or imprecision. Discuss both direction and magnitude of any potential bias	16,17 (338-356)
Interpretation	20	Give a cautious overall interpretation of results considering objectives, limitations, multiplicity of analyses, results from similar studies, and other relevant evidence	15(300-311)
Generalisability	21	Discuss the generalisability (external validity) of the study results	17(355-357)
**Other Information**			

For peer review only - http://bmjopen.bmj.com/site/about/guidelines.xhtml

Funding	22	Give the source of funding and the role of the funders for the present study and, if applicable, for the original study on which the present article is based	19 (407-411)

**Give information separately for cases and controls in case-control studies and, if applicable, for exposed and unexposed groups in cohort and cross-sectional studies.*

Note: An Explanation and Elaboration article discusses each checklist item and gives methodological background and published examples of transparent reporting. The STROBE checklist is best used in conjunction with this article (freely available on the Web sites of PLoS Medicine at http://www.plosmedicine.org/, Annals of Internal Medicine at http://www.annals.org/, and Epidemiology at http://www.epidem.com/). Information on the STROBE Initiative is available at www.strobe-statement.org.

For peer review only

BMJ Open

Linkage of Voluntary Medical Male Circumcision Clients to Adolescent Sexual and Reproductive Health (ASRH) services through Smart-LyncAges project in Zimbabwe: A cohort study

Journal:	*BMJ Open*
Manuscript ID	bmjopen-2019-033035.R3
Article Type:	Original research
Date Submitted by the Author:	02-Feb-2020
Complete List of Authors:	Makoni, Talent; Ministry of Health and Child Care, AIDS and TB Unit ; World Health Organization Regional Office for Africa<br>Thekkur, Pruthu; International Union Against Tuberculosis and Lung Disease, Centre for Operational Research; The Union South-East Asia Office, Centre for Operational Research<br>Takarinda, Kudakwashe ; Ministry of Health and Child Care<br>Xaba, Sinokuthemba; Ministry of Health and Child Care<br>Ncube , Getrude ; Ministry of Health and Child Care<br>Zwangobani, Nonhlahla; Ministry of Health and Child Care<br>Samuelson, Julia; World Health Organization<br>Mangombe, Aveneni; Ministry of Health and Child Care<br>Mabaya, Simbarashe; World Health Organization Regional Office for Africa<br>Tapera, Talent ; Ministry of Health and Child Care<br>Matambo, Ronald ; The Union Zimbabwe<br>Ameyan, Wole ; World Health Organization<br>Mugurungi, Owen; Ministry of Health and Child Care
**Primary Subject Heading**:	Sexual health
Secondary Subject Heading:	Global health, HIV/AIDS, Sexual health, Reproductive medicine, Epidemiology
Keywords:	HIV & AIDS < INFECTIOUS DISEASES, Adolescent Health, SORT IT, Operational Research, Sexual and Reproductive Health, Youth centres

SCHOLARONE™
Manuscripts

I, the Submitting Author has the right to grant and does grant on behalf of all authors of the Work (as defined in the below author licence), an exclusive licence and/or a non-exclusive licence for contributions from authors who are: i) UK Crown employees; ii) where BMJ has agreed a CC-BY licence shall apply, and/or iii) in accordance with the terms applicable for US Federal Government officers or employees acting as part of their official duties; on a worldwide, perpetual, irrevocable, royalty-free basis to BMJ Publishing Group Ltd ("BMJ") its licensees and where the relevant Journal is co-owned by BMJ to the co-owners of the Journal, to publish the Work in this journal and any other BMJ products and to exploit all rights, as set out in our licence.

The Submitting Author accepts and understands that any supply made under these terms is made by BMJ to the Submitting Author unless you are acting as an employee on behalf of your employer or a postgraduate student of an affiliated institution which is paying any applicable article publishing charge ("APC") for Open Access articles. Where the Submitting Author wishes to make the Work available on an Open Access basis (and intends to pay the relevant APC), the terms of reuse of such Open Access shall be governed by a Creative Commons licence – details of these licences and which Creative Commons licence will apply to this Work are set out in our licence referred to above.

Other than as permitted in any relevant BMJ Author's Self Archiving Policies, I confirm this Work has not been accepted for publication elsewhere, is not being considered for publication elsewhere and does not duplicate material already published. I confirm all authors consent to publication of this Work and authorise the granting of this licence.

For peer review only

Linkage of Voluntary Medical Male Circumcision Clients to Adolescent Sexual and Reproductive Health (ASRH) services through Smart-LyncAges project in Zimbabwe: A cohort study

Manuscript Type: Research Article

Short Running Title: Linkage of VMMC clients to ASRH services in Zimbabwe

Investigators and affiliations

Talent M. Makoni[1], Pruthu Thekkur[2,3], Kudakwashe C. Takarinda[4,5], Sinokuthemba Xaba[5], Getrude Ncube[6], Nonhlahla Zwangobani[7], Julia Sumuelson[8], Aveneni Mangombe[9], Simbarashe Mabaya[10], Talent Tapera[11], Ronald Matambo[12], Wole Ameyan[13], Owen Mugurungi[14]

1. National Voluntary Medical Male Circumcision and Adolescent Sexual Reproductive Health Linkages Officer, Ministry of Health, Harare, Zimbabwe

2. Monitoring and Evaluation Officer, Centre for Operational Research, International Union Against Tuberculosis and Lung Diseases, Paris, France

3. The Union-South East Asia Office, New Delhi, India

4. Senior Operational Research Fellow, Centre for Operational Research, The Union, Zimbabwe

5. National Voluntary Medical Male Circumcision Co-ordinator, Ministry of Health, Harare, Zimbabwe

6. National HIV Prevention Co-ordinator, Ministry of Health, Harare, Zimbabwe

7 Director, Technical Services, Zimbabwe National Family Planning Council, Ministry of Health, Harare, Zimbabwe

For peer review only

8. Nurse Epidemiologist, World Health Organization, Geneva, Switzerland

9. National Adolescent Sexual Reproductive Health Co-ordinator, Ministry of Health, Harare, Zimbabwe

10. National Professional Officer, World Health Organization, Zimbabwe

11. Senior Monitoring and Evaluation Officer, Zvandiri Project, AFRICAID, Zimbabwe

12. Operations Officer, The International Union Against Tuberculosis and Lung Diseases, Zimbabwe

13. Technical Officer, Adolescent HIV, World Health Organization, Geneva

14. Director, HIV and TB Programme, Ministry of Health, Harare, Zimbabwe

Corresponding Author:

Talent M. Makoni,

National Voluntary Medical Male Circumcision and Adolescent Sexual Reproductive Health Linkages Officer,

Ministry of Health, Harare, Zimbabwe

Email ID: talent.makoni32@gmail.com

Manuscript details

Word count of abstract - 300; Word count of manuscript - 3482; Tables - 3; Figures - 1; References – 25

For peer review only

ABSTRACT

Objectives: World Health Organization recommended strengthening the linkages between various HIV prevention programmes and Adolescent Sexual Reproductive Health (ASRH) services. The Smart-LyncAges project piloted in Bulawayo city and Mt Darwin district of Zimbabwe established a referral system to link the Voluntary Medical Male Circumcision (VMMC) clients to ASRH services provided at youth centres. Since its inception in 2016, there has been no assessment of the performance of the referral system. Thus, we aimed to assess the proportion of young (10-24 years) VMMC clients getting 'successfully linked' to ASRH services and factors associated with 'not being linked'.

Design: This was a cohort study using routinely collected secondary data.

Setting: All three VMMC clinics of Mt Darwin district and Bulawayo province.

Primary outcome measures: The proportion of 'successfully linked' was summarized as the percentage with a 95% confidence interval (CI). Adjusted relative risks (aRR) using a generalized linear model was calculated as a measure of association between client characteristics and 'not being linked'.

Results: Of 1773 young people registered for VMMC services, 1478 (83%) were referred for ASRH services as they had not registered for ASRH previously. Of those referred for ASRH services, the mean (SD) age of study participants was 13.7 (4.3) years and 427 (28.9%) were out of school. Of the referred, 463 (31.3%, 95% CI- 30.0-33.8) were 'successfully linked' to ASRH services and the median (IQR) duration for linkage was 6 (0-56) days. On adjusted analysis, receiving referral from Bulawayo circumcision clinic (aRR-1.5 (95% CI-1.3-1.7)) and undergoing circumcision at outreach sites (aRR-1.2 (95% CI-1.1-1.3)) were associated with 'not being linked' to ASRH services.

For peer review only - http://bmjopen.bmj.com/site/about/guidelines.xhtml

Conclusion: Linkage to ASRH services from VMMC is feasible as one-third VMMC clients were successfully linked. However, there is need to explore reasons for not accessing ASRH services and take corrective actions to improve the linkages.

Keywords: HIV prevention; Adolescent Health; Sexual and Reproductive Health; Youth centres; SORT IT; Operational Research

Article Summary:

Strengths and Limitations:

- First study to have assessed the extent of linkages with Smart-LyncAges project
- No selection bias as we included all the young people (10-24 years) who registered for circumcision services in the VMMC clinics of two project districts
- Routine programmatic data of large cohort was used and thus reflected the field realities in project implementation
- There might have been an underestimation in the proportion of young people 'successfully linked' to ASRH services as only those who produced referral slip from VMMC clinic were considered as 'successfully linked'
- Study was conducted only during two months of the year and thus might have failed to account for seasonal variations in seeking ASRH services

For peer review only

INTRODUCTION

Young people (aged 10-24 years) are disproportionately affected by the Human Immunodeficiency Virus (HIV) infection, with an estimated 3.9 million living with HIV worldwide in 2017.[1,2] Globally, approximately 1600 youths acquire HIV every day and a youth dies every 10 minutes because of AIDS-related illness.[1] In contrast to the decline in HIV related death rates over the years in other age groups, there has been a rise among young people.[3,4] About 84% of young people living with HIV are in Sub-Saharan Africa and it is estimated that an additional 7.4 million young people might become infected with HIV in Sub-Saharan Africa region alone by 2030.[5]

Unprotected sex is the most common route of HIV infection among young people and is largely due to low knowledge of HIV, early sexual debut, multiple sexual partners, and low condom use.[6] This highlights the need for comprehensive sexuality education (CSE) for adolescents before they become sexually active. Realizing this, the United Nations General Assembly Special Session (UNGASS) on HIV in 2001 recommended access to information, youth-specific HIV education and life skills development to at least 95% of young people.[7] Following this, most Sub-Saharan African countries implemented Adolescent Sexual and Reproductive Health (ASRH) services. However, recent studies from this region have reported poor utilization of ASRH services, with only about 21%-51% of adolescents accessing it.[8,9] Acknowledging the importance and poor utilisation of ASRH services, the World Health Organization (WHO) recommended strengthening the linkages between various HIV prevention and ASRH programmes delivered through general health services.[10]

Zimbabwe, a landlocked Sub-Saharan country, has a high burden of HIV, with an estimated prevalence of 14% among people aged 15 to 49 years.[11] The country has ASRH

For peer review only

indicators showing risky behaviours with about 40% girls and 30% of boys having sex before age eighteen and 62% of males aged 15-24 not using a condom in their last sexual encounter.[12] HIV prevalence in individuals aged 15–19 years was 3.7% and 4.6% for boys and girls, respectively. Among those 20-24 years, the HIV prevalence was 8% and 10.8% for boys and girls, respectively.[12,13] Thus, since 2009, Zimbabwe has implemented Voluntary Medical Male Circumcision (VMMC) and ASRH services as core strategies to limit the HIV burden in young people.

The ASRH programme implemented in 2010, involves the provision of CSE, life skills training, diagnosis and treatment of sexually transmitted infections (STIs) including HIV, family planning counselling, positive masculinity education, vocational training, library services, recreational games and empowerment on rights and responsibility.[14] The ASRH services are delivered at youth-friendly clinics or youth centres. There is anecdotal evidence that the majority of young people do not access ASRH services. Whereas, the VMMC programme implemented as part of an HIV prevention strategy has a high utilization rate.[15] By 2017, about 80% of young boys were covered under VMMC and have had unprecedented contact with the health system. However, the potential of VMMC services as a gateway to additional relevant ASRH services for young people has not been maximized.

The ASRH and VMMC programs have been implemented as two different vertical national health programs, not complementing each other in improving service utilization. In 2016, the WHO supported the Ministry of Health and Child Care to pilot the Smart-LyncAges project to identify approaches to sustain VMMC and improve the complementarity of both services. Through the project, cross-referral mechanisms were established wherein adolescent boys from VMMC are linked to ASRH programmes and vice versa. Though the Smart-LyncAges project has been piloted as a participatory learning approach since 2016,

For peer review only

there is no quantitative information on the extent of successful linkage of clients between VMMC clinics and ASRH services. The information on the extent of successful linkage and factors associated is failure to linkage is necessary to address any deficiencies in the existing referral system prior to scale-up of the project countrywide. In this regard, the study aimed to assess the proportion of young people referred from VMMC clinics successfully linked for ASRH services and factors associated with 'not being linked' in the Smart-LyncAges pilot project sites in Zimbabwe.

For peer review only

METHODOLOGY

Study Design:

This was a cohort study using routinely collected secondary data by the VMMC and ASRH programmes.

Study Setting:

General Setting:

Zimbabwe is a landlocked country in Sub Saharan Africa. The country is divided into ten administrative provinces and has a total of 62 districts. According to the Zimbabwe National Statistics Agency, the country had a projected population of 16,4 million in 2018 and 26% of the total population is adolescents and young adults.[16]

Specific Setting:

The study was conducted in the Bulawayo metropolitan province and Mt Darwin district of Mashonaland Central province, where the VMMC-ASRH linkages project was piloted. Bulawayo is Zimbabwe's second-largest city and has a population of approximately 653,000.[16] Mt Darwin is one of seven districts in the Mashonaland Central province, with a population of about 213,000. In the district, the majority of the population resides in rural areas.

VMMC services and referral to ASRH

The VMMC programme functions under the National AIDS and TB unit with funding support from external sources, mainly The U.S. Presidents Emergency Plan for AIDS Relief (PEPFAR) and Bill and Melinda Gates Foundation (BMGF). The VMMC sites are supported by various non-governmental organizations (NGOs), which act as implementing partners

For peer review only

facilitating service delivery and community mobilization. The Bulawayo Metropolitan province has two VMMC clinics and the Mount Darwin district has one clinic. The circumcision services are provided at static VMMC clinics and also through outreach clinics in the public health facilities.

Demand generation for VMMC services is done through print and mass media advertisements, school health programmes, roadshows and music galas. At the community level, the community mobilizers, peer educators or health workers counsel and refer adolescents and young adults for VMMC clinics. The adolescents and young adults registered for ASRH services are also referred for VMMC. The referral is made to either static VMMC clinics or outreach clinics based on their convenience.

Upon presentation at a VMMC clinic, the clients are registered with a unique VMMC ID number. The clerk documents the details in the 'client intake form'. During registration, the clerk issues the referral slip to all the clients who are not previously registered for ASRH services. The clients are advised to register themselves for ASRH services in their preferred youth centre and referral slip is provided. The referral slip is created in triplicate, one slip given to the adolescent, one maintained in the referral file at the VMMC clinic and the other slip sent to the peer-educator of the service area from where the adolescent has come.

A nurse counsellor offers group education and individual counselling to clients registered for VMMC. During counselling, the importance of registering for ASRH services is emphasized. The nurse counsellors highlight the various ASRH services present in the youth centres and also provide detailing on the benefits of enrolling for ASRH services at youth centres. The nurse counsellors provide HIV testing and a pre-operation examination to assess the eligibility for circumcision surgery. Those adolescents with diabetes, keloids and haemophilia are considered not eligible for surgery. Among those eligible for surgery, either

For peer review only

dorsal slit or forceps guided procedure is performed by either a trained and qualified nurse circumciser or doctor surgeon. Clients are advised to make follow-up visits on day-2 and day-7 after the procedure. All the details on surgery, follow-up and adverse events are recorded in the 'client intake form'.

ASRH services and registration

The ASRH services are provided at the youth centres. The Bulawayo metropolitan province has seventeen youth centres and the Mount Darwin district has two youth centres. Each youth centre is staffed with a youth health advisor (a registered general nurse), a youth facilitator/recreational officer (a social worker) and five peer educators.

Upon arrival at the youth centres, the VMMC clients submit their VMMC-ASRH referral slips. The youth facilitator/recreational officer registers the adolescents for ASRH services and documents the VMMC ID number in the VMMC-ASRH linkages register. The services received by the adolescent during their initial visit are documented in the VMMC-ASRH linkage register. The referral slip received from the adolescent is stored in the referral box maintained at the youth centre.

Study Population:

All adolescents and young adults (10-24 years) registered in VMMC clinics of Mt Darwin district and Bulawayo city during October and November 2018 were included in the study. Those who were previously registered for ASRH services prior to accessing VMMC services were excluded. The sample size was not calculated and there was no sampling as all the adolescents and young adults in both the pilot districts of the Smart-LyncAges project during study reference period were included.

Data Variables, sources of data and data collection:

For peer review only

We extracted details including VMMC identification number, age, education status, mode of referral to VMMC clinic, type of VMMC clinic, HIV status, date of referral to ASRH services, date of registration at VMMC, reason for circumcision, eligibility for circumcision, circumcision status, method of circumcision, status of day-2 post-op visit, status of day-7 post-op visit and adverse events following circumcision within 42 day from the VMMC 'client intake form' maintained at VMMC clinic. The details on registration and date of registration for ASRH services were extracted from the VMMC-ARSH linkage register maintained at youth centres. The information on registration for ASRH services extracted from the VMMC-ASRH register was validated using the referral slips maintained in the referral box of youth centres. Those individuals linked for ASRH services within three months of receiving referral slip were considered as 'successfully linked' to ASRH services.

The data was extracted in March 2019 using two separate structured data extraction proformas designed to extract data from VMMC 'client intake form' and VMMC-ARSH linkage register**.** Thus, for each of the study participants registered during October and November 2018, the linkage status was ascertained only after a minimum of 90 days of follow-up. The principal investigator field-tested data extraction proformas and modified them before data extraction.

For peer review only

Data entry and analysis:

Data were double entered and validated using EpiData Entry software (EpiData Association, Odense, Denmark). Two separate data entry structures were used to enter data from two proformas. The two data structures were then merged using the unique 'VMMC ID number'. The final merged data file was used for analysis.

Data were analyzed using Stata version 12.0 (STATA Corp., College, TX, USA). Socio-demographic, HIV status and clinical characteristics were summarized using percentages. The age of the participants was summarized with mean and standard deviation (SD). The proportion of participant 'successfully linked' to ASRH was summarized as percentages with 95% confidence interval. The duration between referral to 'successfully linked' to ASRH was summarized with median and inter-quartile range (IQR).

The association between socio-demographic and baseline clinical characteristics with 'not being linked' for ASRH service was assessed using bivariate log-binomial regression. A generalized linear model (Poisson regression) with robust variance estimates was used for multivariate regression as the log-binomial model did not converge.[17,18] Initially, all the variables with p value<0.25 in the bivariate model was included in the multivariate model. Later, the variables with a variance inflation factor of more than ten were removed from the final model and adjusted relative risks (aRR) with 95% CI were expressed as measure of association.

Ethics Approval:

The ethics approval was obtained from the Medical Research Council of Zimbabwe (MRCZ/E229) and Ethics Advisory Group of the International Union Against Tuberculosis and Lung Disease, Paris, France (58/18). Permission was sought from the Ministry of Health and Child Care program officials for extracting the routine data collected at VMMC and ASRH centres.

A data sharing statement:

For peer review only

Technical appendix, statistical code, and dataset available from the

https://www.dropbox.com/sh/cm03olkw3qj8j4x/AAArNAzjweJ1iw0UHq86JIFwa?dl=0

Patient and Public Involvement:

Principal Investigator and the data collectors did not interact directly with the young people availing services from VMMC clinics during this retrospective record review. The PI worked with the healthcare staff and peer educators of both the VMMC and ASRH clinics included in the study. Findings from this study will help the Smart-LyncAges project to assess the performance in linkages and also gives insight on deficiencies to be fixed prior to country wide scale-up.

For peer review only

RESULTS

In total, 1773 young people (10-24 years) were registered for VMMC services during the study reference period. Of the total, 1478 (83%) had not been previously registered in the ASRH clinic and were included in the study. The mean (SD) age of study participants was 13.7 (4.3) years and 427 (28.9%) were out of school. Of the 1478 study participants, 1032 (69.8%) were referred for VMMC by community mobilizer and 1230 (83.2%) reported HIV prevention as the reason for seeking circumcision service. Of the total, 6 (0.3%) had HIV infection. The characteristics of study participants are depicted in **Table-1**.

Of the 1478 study participants, 1461 (99%) were eligible for circumcision surgery, of which, 1443 (99%) underwent circumcision (**Figure-1**). Of those who underwent circumcision, 477 (33.1%) had the surgery performed at an outreach site. Of the 1443 participants, 1379 (95.6%) and 1025 (71%) attended day-2 and day-7 review visits, respectively. In total, 20 (1.4%) developed mild adverse events following circumcision and 3 (0.2%) each developed moderate and severe adverse events (**Table-2**).

Of the 1478 study participants referred for ASRH services, 463 (31.3%, 95% CI-29.0%-33.8%) successfully linked for ASRH services at 'youth centre' within three months of referral (**Figure-1**). Among those who were successfully linked, the median (IQR) duration from referral to getting linked at 'youth centre' was 6 (0-56) days. On adjusted analysis, referral from Bulawayo circumcision clinic (aRR-1.5 (95% CI-1.3-1.7)) and undergoing circumcision at outreach sites (aRR-1.2 (95% CI-1.1-1.3)) were associated with not being linked for ASRH services after referral from circumcision clinic. The AIC and BIC of the model were 2799.2 and 2926.3, respectively. The LR test was significant compared to constant model (p value= 0.014). The association between participant's characteristics and not being linked is shown in **Table-3**.

For peer review only

DISCUSSION

This is the first study assessing the extent of successful referral among adolescents and young men between VMMC clinics and ASRH services under the Smart-LyncAges project implemented in Zimbabwe. About 31% of young males (10-24 years) referred from VMMC clinics successfully linked to ASRH services at youth centres within three months of referral. Young people referred from the outreach sites and Bulawayo male circumcision clinic had significantly higher rates of not being linked for ASRH services.

Globally, there is limited literature on linkages between various HIV preventive strategies like VMMC services and ASRH services. A study from Zambia reported that, with enhanced counselling and referral from the community, uptake of ASRH services like HIV testing and counselling, family planning counselling and VMMC services was less than 20% within six months.[19] Also, previous reviews have reported that demand generation activities in the community, improving accessibility and quality of ASRH services, improve ASRH related knowledge but may not increase the utilization of specific ASRH services.[20–22] The potential reason for under-utilization is that the preventive strategy like ASRH may not be considered as an immediate need by young people.[22–24] Considering this, the successful linkage (uptake) of about one in three of the VMMC clients to ASRH services through the Smart-LyncAges project (as they produced referral slips) is promising.

The clients referred from the Bulawayo MC clinic had a slightly higher risk of not being linked to ASRH services. A potential reason for this finding is that the MC clinic is situated in the eastern suburbs of the city where people of high socio-economic status reside. All the fifteen youth centres were located in western suburbs, which are largely populated with people from low socio-economic status. It is possible that the recreational activities available under ASRH services do not appeal to the technologically savvy urban

For peer review only

youths from the elite suburbs. Furthermore, distance to youth centres might have limited the clients from the eastern suburbs to go and register for ASRH services. Similarly, clients accessing VMMC services at outreach clinics were not being linked to ASRH services. The focus of outreach clinics is to conduct surgery in hard to reach areas. Though the referral slips were issued, there would not be space and time to counsel on ASRH services comprehensively. Also, the youth centres in Mt Darwin were mainly located in district centres and might not be accessible to clients of outreach clinics conducted in hard to reach areas. Thus, though not assessed objectively, there might be a rural-urban divide in linkages. Although the actual numbers were small, a high proportion of clients ineligible for circumcision or experiencing an adverse event were referred and successfully linked, suggesting they have additional service needs.

The study had several strengths. First, the study was conducted within the routine programmatic setting, reflecting the field realities and using routine data. Second, all the young males attending the VMMC clinics during the study reference period were included and thus, there was no sampling bias. Third, the study had a large sample size and thus, the estimate of 'successfully linked' was precise. Fourth, the continuous supportive supervision was rendered during the study period to ensure completeness in recording under the project setting and thus, the 'missing data' was limited. Fifth, the registration for ASRH services was validated by cross-checking the stored referral slips along with information extracted from the VMMC-ASRH linkage register and thus, it enhanced the validity of the outcome ascertainment. Sixth, the STrengthening the Reporting of OBservational Studies in Epidemiology (STROBE) guidelines was used to report the study findings.[25]

The study had a few limitations. First, the registration for ASRH services was ascertained based on the VMMC-ASRH linkage register. Only those who produced VMMC

For peer review only

referral slips during registration were listed in the VMMC-ASRH register. Thus, those participants who might have missed their referral slips before registration for ASRH services could have been misclassified as 'not linked'. This could have led to the underestimation of the percentage of 'successfully' linked. Second, the study was conducted only during two months of the year and thus failed to account for seasonal variations in seeking services. During the festive month of December, the youth centres were closed due to holidays. Also, during December, the youth centres in the Mt Darwin region were closed for two weeks due to the cholera outbreak in the region. There might have been an underestimation of 'successfully' linked due to the non-functioning of youth centres during the study follow-up period. However, as there are no estimates of the extent of underestimation, we failed to account for it in estimating the percentage of successfully linked. Third, the pathways for seeking ASRH services and the contribution of peer-educators in reaching the ASRH services were not assessed. Thus, the study failed to document the pathways with the highest successful linkage rate, which could have helped in replicating the model elsewhere. Fourth, the potential confounders like distance from youth centres, socio-economic status, parents' willingness and adolescents' willingness were not captured and included in the adjusted analysis. The inclusion of these variables could have improved the validity of the model. Fifth, the study represents select group of young people who came for VMMC clinics in the project pilot districts. Thus, the generalizability of the study results is limited. Lastly, the study did not explore the reasons for not being linked to ASRH services among those referred.

The study has a few programmatic implications and recommendations. First, the percentage of 'successfully linked' between VMMC clinics and ASRH services was promising. The Smart-LyncAges project can be scaled up to improve the utilization of ASRH services.

For peer review only

However, there is a need for qualitative research to explore the facilitators and barriers to getting linked to ASRH services. This information can help to improve the linkages and effectiveness of the programme.

Second, with the existing records maintained under the project, cohort monitoring of the performance of the referral system was feasible. The 'proportion of those referred from the facility successfully linked for ASRH services' can be introduced as an indicator in the monthly report of each VMMC clinic. This would enable the programme managers to monitor the performance of the referral system better than the absolute number of referred and linked, which is currently being monitored.

Third, the young adolescents seeking VMMC services from outreach clinics at peripheral public health facilities were not getting linked for ASRH services, implying the distance to youth centres was a potential barrier. Solutions should be person-centred, such as co-location of ASRH and VMMC services, improved accessibility with services decentralized to peripheral public health facilities and enhanced training of existing general health staff to deliver youth-friendly services.

Fourth, about 70% of clients came back to avail follow-up services on day-7 post circumcision. This opportunity can be used to educate further and to reinforce the importance of ASRH services. The clients might be more responsive to the information since the circumcision procedure is already completed.

CONCLUSION

The rate of young males referred from VMMC clinics and successfully linked to ASRH services was promising. Those referred from the outreach sites and clinics in Bulawayo had significantly higher rates of 'not being linked'. However, there is a need to explore the

For peer review only

reasons for clients not accessing the ASRH services and take corrective actions to improve the linkages.

ACKNOWLEDGEMENTS

This research was conducted through the Structured Operational Research and Training Initiative (SORT IT), a global partnership led by the Special Programme for Research and Training in Tropical Diseases at the World Health Organization (WHO/TDR). The training model is based on a course developed jointly by the International Union Against Tuberculosis and Lung Disease (The Union) and Medécins sans Frontières (MSF). The specific SORT IT program which resulted in this publication was implemented by the Centre for Operational Research, The Union, Paris, France. Mentorship and the coordination/facilitation of this particular SORT IT workshop was provided through the Centre for Operational Research, The Union, Paris, France; the Department of Tuberculosis and HIV, The Union, Paris, France; The Union, Zimbabwe Office; The Union, South East Asia Office; University of Washington, School of Public Health, Department of Global Health, Seattle, Washington, USA; National Institute for Medical Research, Muhimbili Centre, Dar es Salaam, Tanzania; and AIDS & TB Department, Ministry of Health & Child Care, Harare, Zimbabwe

FUNDING

The training course under which this study was conducted was funded by: the United Kingdom's Department for International Development (DFID); The Global Fund to Fight AIDS, Tuberculosis and Malaria (GFATM) and the World Health Organization (WHO). The WHO also provided funding support for conduct of the study. The funders had no role in

For peer review only

study design, data collection and analysis, decision to publish, or preparation of the manuscript.

AUTHOR CONTRIBUTION

TM was the principal investigator; PT and KT were the SORT IT course mentors; OM, WA, JS, SX and GN were the senior authors. TM, NZ, AM, SM, TT, and RM were involved in data collection; TM, PT and KT analyzed the data and prepared the first draft of the paper. All authors were involved in conception, design, inference of results, providing critical review and approval of the final manuscript.

DISCLOSURE STATEMENT

None of the authors have any competing interests.

For peer review only

REFERENCES

1 Joint United Nations Programme on HIV/AIDS (UNAIDS). Youth and HIV — Mainstreaming a three-lens approach to youth participation. Geneva, Switzerland: 2018.

2 Joint United Nations Programme on HIV/AIDS (UNAIDS). Start Free, Stay Free, AIDS Free. Geneva, Switzerland: 2015.

3 UNICEF. Adolescent deaths from AIDS tripled since 2000 – UNICEF | Press centre | UNICEF. UNICEF. https://www.unicef.org/media/media_86384.html (accessed 8 May 2019).

4 World Health Organization (WHO). WHO | Adolescent deaths and burden of disease. WHO. 2017.https://www.who.int/maternal_child_adolescent/epidemiology/adolescent-deaths-burden-disease/en/ (accessed 8 May 2019).

5 UNICEF. *END AIDS Seventh Stocktaking Report, 2016*. New York, USA: 2016.

6 Joint United Nations Programme on HIV/AIDS (UNAIDS). HIV prevention among adolescent girls and young women. 2016.

7 United Nations General Assembly Special Session on HIV/AIDS. Summary of the Declaration of Commitment on HIV/AIDS - United Nations General Assembly, Special Session on HIV/AIDS, 25-27 June 2001, New York. New York, USA: 2001.

8 Binu W, Marama T, Gerbaba M, *et al.* Sexual and reproductive health services utilization and associated factors among secondary school students in Nekemte town, Ethiopia. doi:10.1186/s12978-018-0501-z

9 Feleke SA, Koye DN, Demssie AF, *et al.* Reproductive health service utilization and associated factors among adolescents (15–19 years old) in Gondar town, Northwest

For peer review only

Ethiopia. *BMC Health Services Research* 2013;**13**:294. doi:10.1186/1472-6963-13-294

10 World Health Organization. The importance of sexual and reproductive health and rights to prevent HIV in adolescent girls and young women in eastern and southern Africa Evidence brief. Geneva, Switzerland: 2017.

11 Ministry of Health and Child Care. ZIMBABWE POPULATION-BASED HIV IMPACT ASSESSMENT ZIMPHIA 2015-16. Harare: 2016.

12 Ministry of Health and Child Care. Zimbabwe Demographic and Health Survey. Harare, Zimbabwe: 2015.

13 Zimbabwe National Statistics Agency. The Zimbabwe Multiple Indicator Cluster. Harae: 2015.

14 Ministry of Health and Child Welfare. National Adolescent Sexual and Reproductive Health Strategy, 2010–2015. Harare, Zimbabwe: 2009.

15 Ministry of Health and Child Care. Accelerated Strategic and Operational Plan 2014-2018. Harare, Zimbabwe: 2019.

16 Zimbabwe National Statistics Agency. ZIMBABWE POPULATION CENSUS 2012. Harare, Zimbabwe: 2012.

17 UCLA Institute for Digital Research and Education. How can I estimate relative risk using glm for common outcomes in cohort studies? | Stata FAQ. Institute for Digital Research and Education. https://stats.idre.ucla.edu/stata/faq/how-can-i-estimate-relative-risk-using-glm-for-common-outcomes-in-cohort-studies/ (accessed 2 Dec 2018).

18 McNutt L-A, Wu C, Xue X, *et al.* Estimating the Relative Risk in Cohort Studies and Clinical Trials of Common Outcomes. *American Journal of Epidemiology* 2003;**157**:940–3. doi:10.1093/aje/kwg074

For peer review only

19 Hewett PC, Nalubamba M, Bozzani F, *et al.* Randomized evaluation and cost-effectiveness of HIV and sexual and reproductive health service referral and linkage models in Zambia. *BMC Public Health* 2016;**16**:785. doi:10.1186/s12889-016-3450-x

20 Mazur A, Brindis CD, Decker MJ. Assessing youth-friendly sexual and reproductive health services: a systematic review. *BMC Health Services Research* 2018;**18**:216. doi:10.1186/s12913-018-2982-4

21 Denno DM, Hoopes AJ, Chandra-Mouli V. Effective Strategies to Provide Adolescent Sexual and Reproductive Health Services and to Increase Demand and Community Support. *Journal of Adolescent Health* 2015;**56**:S22–41. doi:10.1016/J.JADOHEALTH.2014.09.012

22 Chandra-Mouli V, Lane C, Wong S. What Does Not Work in Adolescent Sexual and Reproductive Health: A Review of Evidence on Interventions Commonly Accepted as Best Practices. *Global health, science and practice* 2015;**3**:333–40. doi:10.9745/GHSP-D-15-00126

23 Cassidy C, Bishop A, Steenbeek A, *et al.* Barriers and enablers to sexual health service use among university students: a qualitative descriptive study using the Theoretical Domains Framework and COM-B model. *BMC Health Services Research* 2018;**18**:581. doi:10.1186/s12913-018-3379-0

24 Travers M, O'Uhuru D, Mueller T, *et al.* Implementing Adolescent Sexual and Reproductive Health Clinical Best Practice in the Bronx, New York. *Journal of Adolescent Health* 2019;**64**:376–81. doi:10.1016/j.jadohealth.2018.09.020

25 von Elm E, Altman DG, Egger M, *et al.* The Strengthening the Reporting of Observational Studies in Epidemiology (STROBE) statement: guidelines for reporting observational studies. *The Lancet* 2007;**370**:1453–7. doi:10.1016/S0140-

For peer review only

6736(07)61602-X

For peer review only

505 Table 1: The demographic characteristics, reason for circumcision and HIV status of
506 adolescents and young adults registered for VMMC services during October and
507 November, 2018 in selected health facilities of Zimbabwe, N=1478

Characteristics	Categories	Frequency	(%)
**Total**		**1478**	**(100)**
**Age (in years)**	10-14	980	(66.3)
	15-19	314	(21.2)
	20-24	184	(12.3)
**Education status**	Out of school	427	(28.9)
	Primary education	899	(60.8)
	Secondary education	152	(10.3)
**Health facility**	Mt Darwin	542	(36.7)
	Lobengula MC clinic	587	(39.7)
	Bulawayo MC clinic	349	(23.6)
**Referred by***	Friend or partner	37	(2.5)
	Health worker	216	(14.6)
	Community mobilizer	1032	(69.8)
	Others	139	(9.4)
	Missing	54	(3.7)
**Reasons for circumcision#**	HIV prevention	1230	(83.2)
	Sexual pleasure	248	(16.8)
	STI Prevention	736	(49.8)
	Hygiene	1196	(80.9)
	Socio-cultural reasons	51	(3.5)
**HIV status**	Positive	6	(0.3)
	Negative	1472	(99.7)

510 *Abbreviation:* HIV- Human Immunodeficiency virus; VMMC- Voluntary Medical Male
511 Circumcision; MC- Male Circumcision; STI- Sexually Transmitted Infection
512 * The person who referred the client to VMMC clinic
513 # *Multiple responses are possible*
514
515
516
517
518
519

For peer review only

Table 2: The surgical and follow-up details of adolescents and young adults who underwent circumcision during October and November, 2018 in selected male circumcision clinics of Zimbabwe, N=1443

Characteristics	Categories	Frequency	(%)
**Total**		**1443**	**(100.0)**
**Method of circumcision**	Dorsal slit	1439	(99.7)
	Forceps Guided	4	(0.3)
**Service delivery point***	Static site	966	(66.9)
	Outreach site	477	(33.1)
**Circumciser#**	Nurse practitioner	1418	(98.3)
	Doctor	25	(1.7)
**Attended post-op day-2**	Yes	1379	(95.6)
	No	64	(4.4)
**Attended post-op day-7**	Yes	1025	(71.0)
	No	418	(29.0)
**Adverse event**	None	1417	(98.2)
	Mild	20	(1.4)
	Moderate	3	(0.2)
	Severe	3	(0.2)

* The circumcision in VMMC clinics are considered as static sites. The circumcision done at other public health facilities in a camp mode by staff of VMMC clinics are considered as outreach sites

Circumciser is the one who conducted the circumcision

For peer review only

Table 3: Demographic, HIV status and surgical characteristics associated with not getting linked for ASRH services within 3 months of referral from the selected VMMC health facilities in Zimbabwe, October and November 2018.

Characteristic	Category	Total	Not linked to ASRH, n (%)*	Linked to ASRH, n (%)	Unadjusted RR (95%. CI)	Adjusted RR (95 % CI)[#]	p value
**Total**		**1478**	**1015 (68.7)**	**463 (31.3)**			
**Age (in years)**	10 - 14	980	671 (68.5)	309 (31.5)	1.1 (1.0-1.2)	1.0 (0.9-1.2)	0.828
	15 - 19	314	200 (63.7)	114 (36.3)	1	1	
	20 - 24	184	144 (78.3)	40 (21.7)	1.2 (1.1-1.4)	1.1 (1.0-1.3)	0.168
**Education**	Out of school	427	297 (69.6)	130 (30.4)	1.1 (1.0-1.3)	1.0 (0.9-1.2)	0.560
	Primary	899	622 (69.2)	277 (30.8)	1.1 (1.0-1.2)	1.0 (0.9-1.2)	0.632
	Secondary	152	96 (63.2)	56 (36.8)	1	1	
**Referred By**	Friend/Partner	37	22 (59.5)	15 (40.5)	1	1	
	Health Worker	216	164 (75.9)	52 (24.1)	1.3 (1.0-1.7)	1.2 (0.9-1.6)	0.207
	Community mobiliser	1032	694 (67.3)	338 (32.7)	1.1 (0.9-1.5)	1.1 (0.8-1.5)	0.453
	Others	139	106 (76.3)	33 (23.7)	1.3 (1.0-1.7)	1.0 (0.8-1.4)	0.849
	Missing	54	29 (53.7)	25 (46.3)	0.9 (0.6-1.3)	1.2 (0.8-1.6)	0.345
**Health Facility**	Mt Darwin	542	330 (60.9)	212 (39.1)	1	1	
	Lobengula MC	587	386 (65.8)	201 (34.2)	1.1 (1.0-1.2)	1.1 (1.0-1.3)	0.087
	Bulawayo MC	349	299 (85.7)	50 (14.3)	1.4 (1.3-1.5)	1.5 (1.3-1.7)	<0.001
**HIV Status**	Positive	6	5 (83.3)	1 (16.7)	1.2 (0.8-1.7)	1.3 (0.9-1.9)	0.117
	Negative	1472	1010 (68.6)	462 (31.4)	1	1	
**Reasons for MC**							
HIV Prevention	Yes	1230	865 (85.2)	365 (14.8)	1.2 (1.0-1.3)	1.0 (0.9-1.1)	0.800
	No	248	150 (60.5)	98 (39.5)	1	1	
Sexual Pleasure	Yes	248	158 (63.7)	90 (36.3)	0.9 (0.8-1.0)	0.9 (0.8-1.1)	0.593
	No	1230	857 (69.7)	373 (30.3)	1	1	
STI Prevention	Yes	736	523 (71.1)	213 (28.9)	1.1 (1.0-1.1)	1.0 (0.9-1.1)	0.980
	No	742	492 (66.3)	250 (33.7)	1	1	
Hygiene	Yes	1196	847 (70.8)	349 (29.2)	1.2 (1.1-1.3)	1.1 (1.0-1.2)	0.183
	No	282	168 (59.6)	114 (40.4)	1	1	

For peer review only

Socio-cultural	Yes	51	32 (62.8)	19 (37.2)	0.9 (0.7-1.1)	0.9 (0.7-1.1)	0.407
	No	1427	983 (68.9)	444 (31.1)	1	1	
**Circumcision¥**	Not Eligible	17	3 (17.7)	14 (82.3)	1	1	
	Circumcised	1443	1003 (69.5)	440 (30.5)	3.9 (1.4-11)	2.9 (0.6-8.8)	0.062
	Not circumcised	18	9 (50.0)	9 (50.0)	2.8 (0.9-8.7)	2.3 (0.8-7.1)	0.142
**Circumciser¥**	Nurse	1418	989 (69.8)	429 (30.2)	1.2 (0.9-1.8)	1.2 (0.8-1.8)	0.283
	Doctors	25	14 (56.0)	11 (44.0)	1	1	
**Service delivery point¥**	Static	966	66 (65.8)	900 (34.2)	1	1	
	Outreach	477	367 (76.9)	110 (23.1)	1.2 (1.1-1.2)	1.2 (1.1-1.3)	<0.001
**Day-2 follow-up¥£**	Yes	1379	965 (70.0)	414 (30.0)	1.2 (1.0-1.4)		
	No	64	38 (59.4)	26 (40.6)	1		
**Day-7 follow-up¥**	Yes	1025	732 (71.4)	293 (28.6)	1.1 (1.0-1.2)	0.9 (0.8-1.0)	0.070
	No	418	271 (64.8)	147 (35.2)	1	1	
**Adverse event$¥£**	Yes	26	11 (42.3)	15 (57.7)	1		
	No	1417	992 (70.0)	425 (30.0)	1.7 (1.1-2.6)		

Abbreviations: ASRH-Adolescent Sexual Reproductive Health; STI-Sexually Transmitted Infection; HIV-Human Immunodeficiency Virus; RR-Relative Risk, CI-Confidence Interval

* Row percentage; # Using generalized linear model (Poisson regression); $ Mild, Moderate and Severe were clubbed as 'Yes'; ¥ Applicable only for those who underwent circumcision; £ These factors were removed from the final model as they had variance inflation factor of more than 10

For peer review only

548 **Figure-1**: Flow-chart depicting the adolescents eligible for referral to ASRH services, circumcision status and linkage to ASRH services among
549 those registered at selected VMMC clinics of Zimbabwe during October to November 2018

550

551 Abbreviation: VMMC- Voluntary Medical Male Circumcision; ASRH- Adolescent Sexual Reproductive Health

552

For peer review only

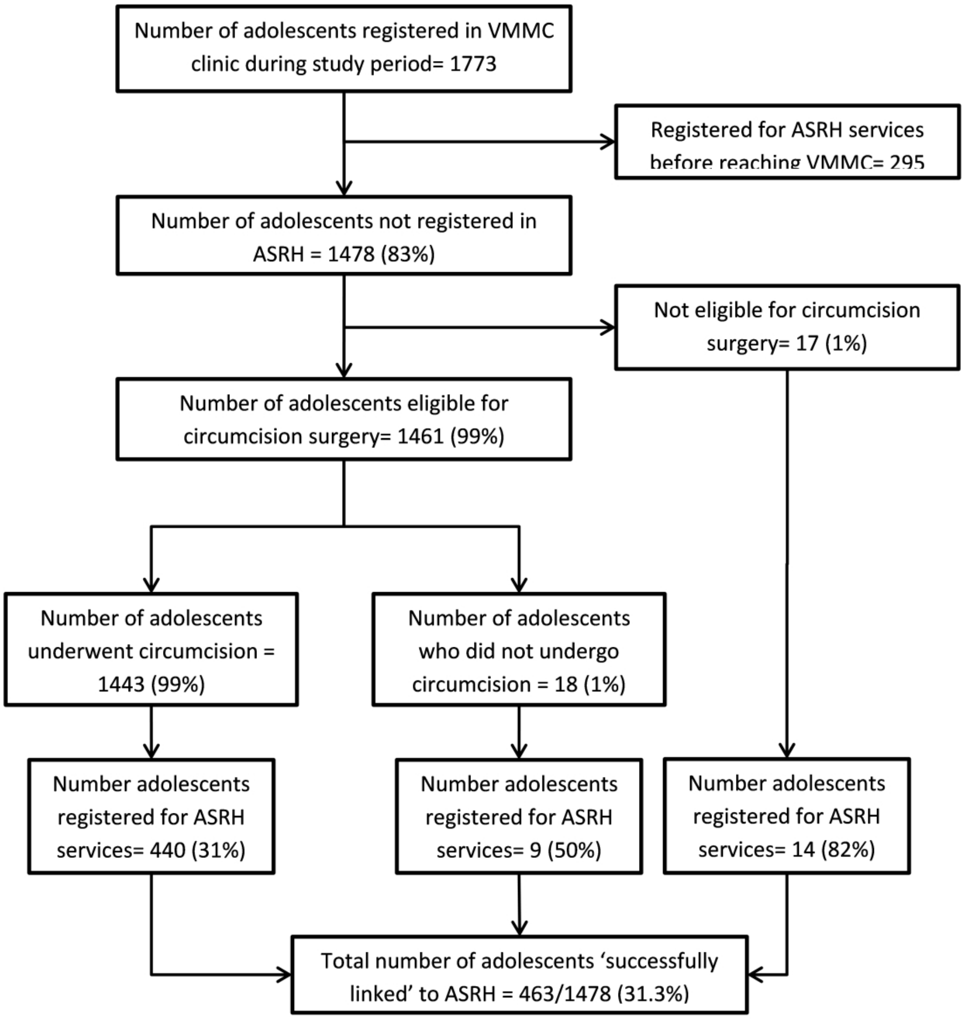

Figure-1: Flow-chart depicting the adolescents eligible for referral to ASRH services, circumcision status and linkage to ASRH services among those registered at selected VMMC clinics of Zimbabwe during October to November 2018

Abbreviation: VMMC- Voluntary Medical Male Circumcision; ASRH- Adolescent Sexual Reproductive Health

1123x1193mm (96 x 96 DPI)

STROBE Statement

Checklist of items that should be included in reports of observational studies

Section/Topic	Item No	Recommendation	Reported on Page No (Line No)
**Title and abstract**	1	(*a*) Indicate the study's design with a commonly used term in the title or the abstract	1(2-3)
		(*b*) Provide in the abstract an informative and balanced summary of what was done and what was found	3(38-67)
**Introduction**			
Background/rationale	2	Explain the scientific background and rationale for the investigation being reported	5-7(85-145)
Objectives	3	State specific objectives, including any prespecified hypotheses	7(142-145)
**Methods**			
Study design	4	Present key elements of study design early in the paper	8(149-150)
Setting	5	Describe the setting, locations, and relevant dates, including periods of recruitment, exposure, follow-up, and data collection	8-10(153-210)
Participants	6	(*a*) *Cohort study*—Give the eligibility criteria, and the sources and methods of selection of participants. Describe methods of follow-up<br>*Case-control study*—Give the eligibility criteria, and the sources and methods of case ascertainment and control selection. Give the rationale for the choice of cases and controls<br>*Cross-sectional study*—Give the eligibility criteria, and the sources and methods of selection of participants	10(213-216)
		(*b*) *Cohort study*—For matched studies, give matching criteria and number of exposed and unexposed<br>*Case-control study*—For matched studies, give matching criteria and the number of controls per case	
Variables	7	Clearly define all outcomes, exposures, predictors, potential confounders, and effect modifiers. Give diagnostic criteria, if applicable	11(220-230)
Data sources/measurement	8*	For each variable of interest, give sources of data and details of methods of assessment (measurement). Describe comparability of assessment methods if there is more than one group	11(231-234)
Bias	9	Describe any efforts to address potential sources of bias	11(237-240, 245-249)
Study size	10	Explain how the study size was arrived at	10(213-216)
Quantitative variables	11	Explain how quantitative variables were handled in the analyses. If applicable, describe which groupings were chosen and why	11,12(241-244)
Statistical methods	12	(*a*) Describe all statistical methods, including those used to control for confounding	11,12(241-249)
		(*b*) Describe any methods used to examine subgroups and interactions	
		(*c*) Explain how missing data were addressed	11(229-230)

For peer review only - http://bmjopen.bmj.com/site/about/guidelines.xhtml

		(*d*) *Cohort study*—If applicable, explain how loss to follow-up was addressed<br>*Case-control study*—If applicable, explain how matching of cases and controls was addressed<br>*Cross-sectional study*—If applicable, describe analytical methods taking account of sampling strategy	NA
		(*e*) Describe any sensitivity analyses	

Section/Topic	Item No	Recommendation	Reported on Page No(Line No)
**Results**			
Participants	13*	(a) Report numbers of individuals at each stage of study—eg numbers potentially eligible, examined for eligibility, confirmed eligible, included in the study, completing follow-up, and analysed	14(277-280)
		(b) Give reasons for non-participation at each stage	
		(c) Consider use of a flow diagram	Figure-1
Descriptive data	14*	(a) Give characteristics of study participants (eg demographic, clinical, social) and information on exposures and potential confounders	14(270-276)
		(b) Indicate number of participants with missing data for each variable of interest	
		(c) *Cohort study*—Summarise follow-up time (eg, average and total amount)	14(283-286)
Outcome data	15*	*Cohort study*—Report numbers of outcome events or summary measures over time	14(283-284)
		Case-control study—Report numbers in each exposure category, or summary measures of exposure	
		Cross-sectional study—Report numbers of outcome events or summary measures	
Main results	16	(*a*) Give unadjusted estimates and, if applicable, confounder-adjusted estimates and their precision (eg, 95% confidence interval). Make clear which confounders were adjusted for and why they were included	14(286-290)
		(*b*) Report category boundaries when continuous variables were categorized	26 (525)
		(*c*) If relevant, consider translating estimates of relative risk into absolute risk for a meaningful time period	
Other analyses	17	Report other analyses done—eg analyses of subgroups and interactions, and sensitivity analyses	
**Discussion**			
Key results	18	Summarise key results with reference to study objectives	15(294-299)
Limitations	19	Discuss limitations of the study, taking into account sources of potential bias or imprecision. Discuss both direction and magnitude of any potential bias	16,17 (338-356)
Interpretation	20	Give a cautious overall interpretation of results considering objectives, limitations, multiplicity of analyses, results from similar studies, and other relevant evidence	15(300-311)
Generalisability	21	Discuss the generalisability (external validity) of the study results	17(355-357)
**Other Information**			

For peer review only - http://bmjopen.bmj.com/site/about/guidelines.xhtml

Funding	22	Give the source of funding and the role of the funders for the present study and, if applicable, for the original study on which the present article is based	19 (407-411)

**Give information separately for cases and controls in case-control studies and, if applicable, for exposed and unexposed groups in cohort and cross-sectional studies.*

Note: An Explanation and Elaboration article discusses each checklist item and gives methodological background and published examples of transparent reporting. The STROBE checklist is best used in conjunction with this article (freely available on the Web sites of PLoS Medicine at http://www.plosmedicine.org/, Annals of Internal Medicine at http://www.annals.org/, and Epidemiology at http://www.epidem.com/). Information on the STROBE Initiative is available at www.strobe-statement.org.

For peer review only
